# Ionic Strength‐Induced Compartmentalization for Nanogel‐in‐Microgel Colloids

**DOI:** 10.1002/smll.202410221

**Published:** 2025-01-15

**Authors:** Maria I. Pieper, Hannah F. Mathews, Andrij Pich

**Affiliations:** ^1^ DWI‐Leibniz Institute for Interactive Materials e.V. RWTH Aachen University Forckenbeckstr. 50 52074 Aachen Germany; ^2^ Institute of Technical and Macromolecular Chemistry RWTH Aachen University Worringerweg 2 52074 Aachen Germany; ^3^ Aachen Maastricht Institute for Biobased Materials (AMIBM) Maastricht University Brightlands Chemelot 6167 RD Geleen The Netherlands

**Keywords:** aggregation, colloids, gels, polymers

## Abstract

Compartmentalization is crucial for control over complex biological cascade reactions. In microgels, the formation of discrete compartments allows for simultaneous uptake and orthogonal release of physicochemically distinct drugs, among others. However, many state‐of‐the‐art approaches yielding compartmentalized microgels require the use of specific, though not always biocompatible, components and temperatures well above the physiological range, which may damage possible biological cargo. Therefore, a novel technique to fabricate compartmentalized microgels by exploiting ionic strength‐induced precipitation as a mechanism for compartmentalization is developed. For this, a droplet‐based microfluidic approach in which preformed nanogels are incorporated into poly(*N*‐isopropylacrylamide)‐ or poly(acrylamide)‐based microgels is employed. Allowing contact between the nanogel–monomer mixture and a salt solution only at the cross junction inhibits premature precipitation of the nanogels and aggregates form on the chip. It is demonstrated that this method is applicable to a variety of nanogel species in both stimuli‐responsive and non‐stimuli‐responsive microgel networks. For temperature‐responsive nanogel compartments in non‐responsive microgels, anisotropic shape change is investigated by adjusting temperature or salt concentration or changing the solvent. Lastly, an exemplary uptake and release experiment demonstrates highly selective drug absorption, paving the way for more advanced biomimetic polymer structures.

## Graphical abstract

The ionic strength‐induced compartmentalization is a facile and robust method to precipitate particles such as charged or uncharged nanogels inside aqueous microfluidic droplets prior to polymerization. The use of different materials for the compartment and the surrounding network can facilitate anisotropic shape change of the resulting colloids. This method opens new pathways for compartmentalized particles for applications like drug delivery.

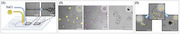

## 1 Introduction

Three‐dimensionally crosslinked colloidal polymer networks can swell in a good solvent like water and mimic biological tissue. These micro‐sized gels are categorized into “microgels” with diameters between 0.1 and 100 µm and “nanogels” in a size range of 1 to 100 nm,^[^ ^1^ ^]^ though oftentimes the boundaries are less clear and authors use these terms less strictly. The polymer network itself can be of synthetic origin^[^ ^2^ ^]^ or bio‐based.^[^ ^3^ ^]^ Depending on the specific chemical composition, their physicochemical properties vary greatly and can be finely tuned for specific applications. Additional properties of nano‐ and microgels include a porous structure and possible stimuli‐responsiveness. Such stimuli‐responsive microgels can shrink or swell upon exposure to external stimuli such as temperature,^[^ ^4^, ^5^ ^]^ pH value,^[^ ^6^ ^]^ or UV light^[^ ^5^, ^7^ ^]^ depending on the chemical composition. Consequently, microgels find application in a variety of fields including catalysis,^[^ ^8^ ^]^ sensors,^[^ ^9^ ^]^ and drug delivery systems.^[^ ^10^, ^11^ ^]^

Compartmentalization is a desirable feature for microgels as it was shown to increase the efficacy of drug carriers.^[^ ^12^, ^13^ ^]^ In addition, phase separation in microgels allows for the incorporation of multiple drugs or molecules with opposing properties that otherwise cannot be incorporated into one carrier. This also prevents opposing functionalities from diminishing their effects.^[^ ^12^ ^]^ In catalysis, compartments in microgels facilitate the implementation of reactions, such as enzymatic cascades.^[^ ^11^, ^14^ ^]^ Lastly, anisotropy combined with functional compartments is key for the design of microbots and microswimmers.^[^ ^15^ ^]^

Compartmentalized microgels have been synthesized before through various techniques, one of the most prominent ones being precipitation polymerization. Besides core–shell microgels,^[^ ^16^ ^]^ more complex compartmentalized microgels were produced by mixing polyanionic and polycationic reactive precursor solutions at different reaction times to obtain Janus‐like microgels through coacervation.^[^ ^17^ ^]^ In another approach, precipitation polymerization was employed to synthesize microgels, which were in turn used to seed another polymerization in a cascade flow process to yield raspberry‐like patchy particles.^[^ ^18^ ^]^ Raspberry‐like microgels can also be prepared by employing self‐assembling comonomers in a semi‐batch precipitation polymerization, which can also yield core–shell or dumbbell‐like microgels by adjusting the comonomer content.^[^ ^19^ ^]^ However, it is difficult to control microgel morphology with such methods, which is why intensive optimization^[^ ^17^ ^]^ or support by computer simulations^[^ ^19^ ^]^ are often required.

The droplet‐based microfluidic approach offers several advantages including the fabrication of highly monodisperse droplets,^[^ ^20^, ^21^ ^]^ the need for only low reaction volumes and facile automation,^[^ ^22^ ^]^ as well as precise control over flows^[^ ^20^ ^]^ and therefore simple adjustment of microgel compositions. Droplet‐based microfluidics has also been used for the fabrication of compartmentalized microgels before. For instance, Zhang et al. have used flow‐focusing microfluidics with two, three, or four different aqueous phases containing both alginate and a calcium‐ethylenediaminetetraacetic acid (EDTA) complex to form droplets emulsified in oil at the flow‐focusing cross junction. In the laminar flow regime, mixing was avoided until acid was introduced through the oil phase at a second cross junction. The acid then induced the release of calcium ions from EDTA after diffusion into the aqueous droplets. Consequently, the alginate was crosslinked by free calcium ions to yield microgels with two, three, or four spatially separated compartments containing either fluorescein‐ or rhodamine‐labeled nanoparticles.^[^ ^23^ ^]^ Calcium alginate gelation has also been used in a double emulsion approach to form dumbbell‐shaped microgels with two distinct compartments. For that, two aqueous solutions containing alginate, complexed calcium, and different dyes were separately dispersed in an acidic oil phase at a cross junction so that distinct droplets of each solution formed. Subsequently, at a second cross junction, the water‐in‐oil emulsion was dispersed in another continuous water phase. This way, oil droplets comprising two different water droplets formed. As the acid slowly diffused from the surrounding oil into the internal water droplets, the water droplets started to coalesce whilst the alginate gelled, which resulted in Janus‐like microgel structures.^[^ ^24^ ^]^

In contrast to these multi‐phase and multi‐emulsion approaches, compartmentalization in microgels can further be realized by phase separation from homogeneous droplets. For this, microfluidic droplets are formed that contain responsive materials, which in turn change their properties (e.g., they become hydrophobic) upon exposure to specific stimuli and thus aggregate on one side of the droplet. Examples of this include droplets that contain light‐sensitive random copolymers that phase‐separate by UV irradiation^[^ ^25^ ^]^ as well as compartmentalized microgels with agarose, poly(ethylene glycol), and dextran that were obtained through solvent evaporation.^[^ ^26^ ^]^ Though many stimuli can be employed for phase separation and thus, compartmentalization, this method is limited to specific responsive materials.

In most examples, temperature‐responsive materials are employed for phase separation^[^ ^27^ ^]^ from homogeneous droplets.^[^ ^21^, ^28^ ^]^ For instance, in the work of Shah et al., cationic p(*N*‐isopropylacrylamide) (PNIPAAm)/polyallylamine microgels and polyacrylic acid were dispersed in droplets containing the monomer acrylamide (AAm), a crosslinker and a photoinitiator. Droplets formed from this solution would undergo phase separation at high temperatures as the thermo‐responsive PNIPAAm‐based microgels collapsed, precipitated, and agglomerated on one side of the microgel together with the polyanions as a result of electrostatic interactions. Polymerization of these droplets by activation with UV light yielded Janus‐like particles with an acrylamide‐ and a microgel‐rich side.^[^ ^21^ ^]^ The drawback of this method is not only that it is limited to thermo‐responsive components, but also that temperature‐sensitive biological cargo cannot be incorporated using this method.

Another trigger used for the induction of phase separation in aqueous droplets is ionic strength. Homogeneous water droplets containing alginate and iron oxide nanoparticles were dispersed in high‐viscosity oil and placed on an agarose gel that contained high amounts of salt. Due to the resulting salt concentration gradient between the agarose gel and the water droplets, salt diffused from the agarose gel into the droplets, while water diffused from the droplets into the agarose gel. Thus, the salt concentration in the water droplets increased and the iron oxide nanoparticles agglomerated on one side of the droplets, which sustained shrinkage gelation to yield Janus‐like microgels with an iron‐rich side.^[^ ^29^ ^]^ However, this technique requires multiple steps, takes a long time as it is a non‐continuous batch approach, and is only applicable for certain droplet compositions that allow for this specific type of gelation. Additionally, the exact ionic strength present in the droplets cannot be controlled this way. Current challenges include finding a controllable and easy‐to‐implement way of using ionic strength for compartmentalization as it poses several advantages compared to temperature‐induced phase separation including milder conditions.

Lastly, in our previous research, we successfully synthesized microgels with compartments consisting of both negatively and positively charged polyelectrolyte nanogels, terming them nanogel‐in‐microgel colloids (NiM‐C). In principle, we prepared dispersions that contained both types of nanogels, as well as NIPAAm, a crosslinker, and a photoinitiator for the production of microfluidic droplets. We could show that the combination of the electrostatic attraction between the oppositely charged polyelectrolyte nanogels and the depletion flocculation that occurred upon the generation of linear and crosslinked polymer chains during the UV irradiation led to the formation of polyampholyte compartments.^[^ ^30^ ^]^ Nonetheless, the very specific combination of positively and negatively charged nanogels limits the applicability of this approach to other systems such as polyelectrolyte or uncharged nanogels, or other particles.

To circumvent these restrictions, we present a one‐step ionic strength‐induced compartmentalization approach performed in droplet‐based microfluidics. In this work, uncharged as well as either positively or negatively charged PNIPAAm‐based polymer gels were synthesized by precipitation polymerization to yield gels with hydrodynamic diameters of ≈500 nm. To distinguish them from microgels prepared in droplet‐based microfluidics, which are several orders of magnitude larger, they are referred to as “nanogels” here for clear differentiation. Subsequently, for synthesis and purification, the different nanogel species were incorporated into thermo‐responsive (NIPAAm) and non‐temperature responsive (AAm) microgels at varying ionic strengths. Herein, the term “nanogel‐in‐microgel colloids” (NiM‐C) refers to microgels containing smaller nanogels.

The novel and optimized synthesis route is presented and the broad applicability is demonstrated with nanogels that are uncharged, positively and negatively charged in both PNIPAAm‐ and PAAm‐based microgels. The nanogels were fluorescently labeled to enable localization inside the NiM‐C using confocal laser scanning microscopy (CLSM) to verify that adjusting the ionic strength during synthesis allows for precise control over nanogel localization and compartmentalization. In addition, the method is also applied to metal nanoparticles to produce metal nanoparticle compartments in microgels. For PAAm‐based NiM‐C, which consist of thermo‐responsive nanogel compartments in a non‐responsive matrix, anisotropic shape change of the NiM‐C is investigated regarding both changes in compartment size and changes in surrounding matrix size. Next, permeability assays were performed in CLSM to examine the influence of compartmentalization and salt on microgel permeability. Lastly, a proof‐of‐principle experiment exemplifies the successful and highly selective uptake of a model drug into the compartments and its subsequent release.

## 2 Results and Discussion

### 2.1 Synthesis of NiM‐C via Ionic Strength‐Induced Compartmentalization

Polymers as well as colloidal polymer networks can exhibit responsiveness toward external stimuli depending on their chemical composition. PNIPAAm, for instance, is a polymer that combines both hydrophilic and hydrophobic parts with its amide and isopropyl groups, respectively. Consequently, PNIPAAm exhibits thermo‐responsive properties: it is dissolved below its lower critical solution temperature (LCST) of 32 °C^[^ ^31^ ^]^ as the polymer is hydrated, while above the LCST the added thermal energy induces fluctuations in this hydration. This leads to water molecules leaving the polymer, which causes the polymer to change into a globular state and precipitate.^[^ ^32^ ^]^ In the case of crosslinked polymers or polymeric colloids, this phenomenon is referred to as volume phase transition temperature (VPTT). Besides thermo‐responsiveness, PNIPAAm also reacts to changes in ionic strength. At low ionic strengths, PNIPAAm chains are hydrated through the formation of hydrogen bonds between polymer segments and water molecules. However, with an increase in ionic strength, the added electrolytes compete with the polymer chains for the hydration by water molecules, and the number of hydrogen bonds between the polymer segments and water molecules is reduced. At high enough ionic strengths, the hydrophobic interactions between polymer segments dominate over hydrogen bonding and microgels deswell (Figure S2A, Supporting Information).^[^ ^33^ ^]^ Above a critical salt concentration, the electrolyte–water interactions and hydrophobic interactions between polymer chains dominate and microgels form aggregates, which can precipitate from aqueous solution. This effect is visible even macroscopically for a variety of PNIPAAm‐based nanogels (Figure S1, Supporting Information).

This effect is exploited for the ionic strength‐induced phase separation in aqueous microfluidic droplets. Uncharged PNIPAAm‐based nanogels were chosen as thermo‐responsive model particles. For the larger microgel network, both PNIPAAm and PAAm were used. The incorporation of PNIPAAm nanogels in PNIPAAm microgels is already established^[^ ^30^ ^]^ and enables direct comparison between both methods. PAAm is a material that is non‐responsive and thus, in combination with the thermo‐responsive nanogels, facilitates anisotropic shape change behavior.

The uncharged PNIPAAm‐based nanogels were synthesized in a semi‐batch approach, where a solution containing NIPAAm, *N*,*N′*‐methylenebis(acrylamide) (BIS), hydrochloric acid and sodium dodecyl sulfate (SDS) was initiated with 2,2′‐azobis[*N*‐(2‐carboxyethyl)2‐methylpropionamidine] (ACMA) (Figure S3, Supporting Information). Methacryloxyethyl thiocarbamoyl rhodamine B (RB) was added belatedly as a fluorescent comonomer for later visualization in CLSM. The exact amounts of the used chemicals can be found in Table S1 (Supporting Information).

**Figure 1:**
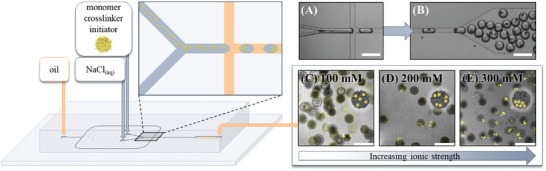
Schematic depiction of the droplet‐based microfluidic setup. One aqueous phase contains a monomer, crosslinker, initiator, and nanogels, the other consists of a sodium chloride solution. A) At the interface where the two water phases come in contact, the locally high salt concentration induces the collapse and precipitation of the nanogels. B) After water‐in‐oil droplet formation at the cross junction, phase‐separated compartments form inside the droplets. C–E) CLSM images show PNIPAAm‐based NiM‐C with varying degrees of compartmentalization depending on the ionic strength used during synthesis, which is exemplary illustrated by the schematics inserted into the respective images. Scale bars represent 300 µm.

After purification via dialysis, the lyophilized nanogels were redispersed in HPLC‐grade water at the desired concentrations for droplet‐based microfluidic synthesis. For this, NIPAAm or AAm, the crosslinker BIS, and the photoinitiator lithium phenyl‐2,4,6‐trimethylbenzoylphosphinate (LAP) (amounts given in Tables S2 and S3, Supporting Information) were dissolved in a nanogel dispersion to be used as the first aqueous phase. A sodium chloride solution at the desired concentration served as a second aqueous phase. Here, it is important to consider that both aqueous phases were employed at a flow ratio, and therefore a volume ratio, of 1:1. Thus, the concentration of the components is halved after water‐in‐oil droplet formation on a chip. Hydrofluoroether (HFE) oil with 2 wt.% of the neutral surfactant FluoSurf was used for the oil phase to stabilize the emulsion and avoid unwanted electrostatic interactions between the surfactant and charged components present in the water phase. At high enough ionic strengths, the nanogels precipitate at the cross junction where the two aqueous phases meet and form compartments (Figure S4, Supporting Information). The water‐in‐oil emulsion was collected in a vial and irradiated with UV light for 10 s to initiate the polymerization. This approach is schematically shown in **Figure** **1** (and Figure S3, Supporting Information).

The CLSM images in Figure 1 exemplary verify the successful incorporation of uncharged nanogels (shown in yellow) into PNIPAAm‐based NiM‐C (dark grey) and precise control over compartmentalization. For uncharged nanogels incorporated at 100 mm (Figure 1C), an overall homogeneous nanogel distribution with only ≈10% of microgels exhibiting compartments (Table S4, Supporting Information) can be observed, while for 200 mm, larger loose compartments form in ≈50% of the microgels (Table S4, Supporting Information), as illustrated by the schematics (Figure 1D). Above, at 300 mm, phase‐separated and distinct compartments are immobilized inside the microgel network (Figure 1E). The precise adjustment of the ionic strength present in the droplets allows for exact control over nanogel distribution inside NiM‐C. This is a decisive advantage over the current use of ionic strength for precipitation of particles inside droplets, where control over ionic strength was not possible.^[^ ^29^ ^]^ Moreover, the ionic strength can easily and rapidly be adjusted during microfluidic synthesis by changing the second aqueous phase that contains sodium chloride. This way, different salt concentrations can be screened in an efficient manner to optimize the synthesis. The ionic strength must be high enough to precipitate nanogels, but low enough to avoid clogging the channel.

Overall, the ionic strength‐induced compartmentalization in droplet‐based microfluidics presented here was shown to be a simple and precisely adjustable tool for the production of microgels with distinct nanogel compartments.

To test the tolerance and transferability of this technique, we also employed AAm as a monomer for the production of NiM‐C and incorporated a variety of PNIPAAm‐based nanogels. In total, we immobilized uncharged, negatively charged, and positively charged nanogels as well as the combination of negatively and positively charged nanogels in both PNIPAAm‐ and PAAm‐based NiM‐C.

The polyelectrolyte PNIPAAm‐based nanogels were synthesized as described before.^[^ ^30^ ^]^ Briefly, for the positively charged nanogels, NIPAAm, BIS, and *N*‐(3‐Aminopropyl)‐methacrylamide (AMPH) were dissolved in HPLC‐grade water. The pH value was adjusted to basic conditions suitable for the synthesis with the addition of sodium hydroxide solution. The solution was degassed with nitrogen and heated to 70 °C before a 2,2′‐azobis(2‐methylpropionamidine) (AMPA) solution was added to initiate the reaction. The synthesis was terminated after 4 h. After dialysis, the positively charged nanogels were fluorescently labeled with Cyanine5‐NHS ester for visualization in CLSM. The negatively charged nanogels were synthesized similarly to the uncharged nanogels with the addition of methacrylic acid (MAAc) to the monomer solution.

**Figure 2:**
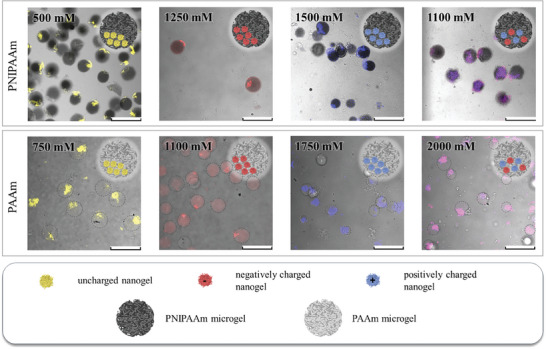
Confocal laser scanning microscopy overlay images of PNIPAAm‐ (top, dark grey) and PAAm‐based NiM‐C (bottom, light grey) containing uncharged nanogels (yellow), negatively charged nanogels (red), positively charged nanogels (blue) and a mixture of negatively and positively charged nanogels (pink). The ionic strengths contained in the aqueous droplets during microfluidic synthesis are denoted in the images. PAAm‐based NiM‐C is marked for better visibility due to the similar refractive index of PAAm and water. Scale bars represent 300 µm.

**Figure** **2** shows the compartmentalized NiM‐C containing uncharged nanogels (yellow), negatively charged nanogels (red), positively charged nanogels (blue), and the combination of the charged nanogels (overlay of red and blue shown in pink) for both PNIPAAm‐ (dark grey) and PAAm‐based microgel networks (light grey). For all investigated microgel networks and nanogel combinations, the production of compartmentalized NiM‐C was successful. In nearly all samples, the compartments are very distinct and mostly located at the periphery of the gel. A possible explanation for the localization of the compartments inside the microgels is the gravitational forces acting on the denser nanogel agglomerates inside the aqueous droplets before UV irradiation and polymerization. Interestingly, in PNIPAAm NiM‐C, uncharged nanogels form compartments at 300 mm, as seen in Figure 1, while the compartmentalization of charged nanogels requires salt concentrations above 1000 mm in all cases. This trend can also be seen in PAAm‐based samples: Uncharged nanogels precipitate at only 750 mm, while negatively and positively charged nanogels form compartments at 1100 and 1750 mm, respectively (Figure 2). For nearly all samples, 100% of the microgels are compartmentalized, while in PNIPAAm‐based NiM‐C containing positively charged nanogels and in PAAm‐based NiM‐C containing negatively charged nanogels only ≈50% and 40% of microgels, respectively, are compartmentalized. In these samples, some microgels still contain dispersed nanogels or only small amounts of nanogels due to precipitating nanogels clogging the microfluidic channel, which is why a further increase of the salt concentration does not yield better compartmentalization. The reason for the different ionic strengths required for compartmentalization lies in the different mechanisms for the precipitation. The precipitation of uncharged PNIPAAm‐based nanogels is caused by the competition of added electrolytes with polymer chains for hydration by water molecules.^[^ ^33^ ^]^ In the case of charged nanogels, the added salt first screens the charges that are responsible for the swelling of the nanogels due to electrostatic repulsion and osmotic pressure until hydrophobic interactions dominate and the nanogels collapse (Figure S2A,B, Supporting Information). Additionally, the charged nanogels are more hydrophilic than the uncharged PNIPAAm nanogels and interact more strongly with water molecules; therefore, higher ionic strength is necessary for the electrolyte–water interactions to dominate and the charged nanogels to precipitate.

Moreover, the CLSM images indicate a co‐precipitation of the charged nanogels. While the negatively charged nanogels precipitate at ≈1250 mm and the positively charged nanogels precipitate at ≈1500 mm, the combination of the two exhibits simultaneous precipitation, as seen in Figure 2. A more detailed study of the co‐precipitation is presented in Figure S5 (Supporting Information). Since the nanogels are dispersed and fully swollen in water, their loose chain ends may entangle before the nanogels collapse and precipitate, which could cause the two nanogel species to precipitate together. It is important to note that the ionic strength is high enough to completely screen all charges,^[^ ^30^ ^]^ ruling out electrostatic attraction as the cause for the observed co‐precipitation. The same effect can be observed in PAAm‐based samples. Overall, all investigated nanogel species could be used for the formation of compartments in PNIPAAm and PAAm NiM‐C, which is not possible with other approaches that, for instance, require electrostatic attraction between nanogel species.^[^ ^30^ ^]^

As further proof of the broad applicability of the introduced method, PNIPAAm‐ and PAAm‐based microgels with compartmentalized iron oxide nanoparticles were fabricated using the same technique (Figure S6, Supporting Information). In other approaches, compartmentalization of metal nanoparticles in microgels is either more complicated as it is not achieved from homogeneous droplets^[^ ^34^ ^]^ or the method does not offer precise control over the ionic strength present in the droplets.^[^ ^29^ ^]^

These results show that the ionic strength‐induced compartmentalization is highly versatile and applicable for many different nanogel and microgel compositions as well as metal nanoparticles with high control over the degree of compartmentalization. For the combination of multiple nanogel species, co‐precipitation must be considered and for all systems, optimization is required to obtain compartmentalized colloids while reducing clogging during the microfluidic synthesis. This method can be employed for the production of compartmentalized microgels with a variety of different properties designed for many possible applications.

### 2.2 Characterization of NiM‐C Containing Uncharged Nanogels

In the following, the characterization focuses on NiM‐C containing uncharged nanogels only. Both the anisotropic shape change behavior as well as the permeability assays are representative of all types of nanogels employed in this work because the polyelectrolyte nanogels only contain 10 mol% of the respective comonomers, and the hydrodynamic radii are very similar for all nanogel species. Thus, we assume the charges will have a negligible influence on NiM‐C properties like thermo‐responsiveness and permeability.

#### 2.2.1 Stimuli‐Responsiveness and Anisotropic Shape Change

One of the most interesting characteristics of phase‐separated particles is their ability to combine multiple (contrasting) physicochemical properties. The compartmentalized PAAm NiM‐C unites a more hydrophobic as well as thermo‐ and ionic strength‐responsive PNIPAAm‐based nanogel compartment and a highly hydrophilic and non‐responsive PAAm microgel compartment.

**Figure 3:**
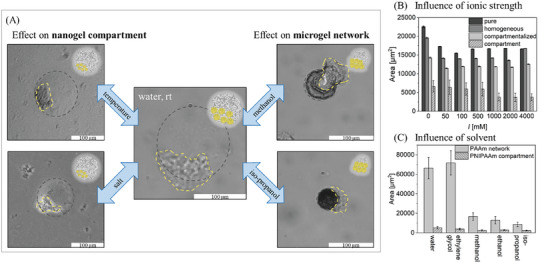
A) Overview of anisotropic shape change behavior of PAAm‐based NiM‐C upon change in temperature, ionic strength, or polarity of solvent depicted by microscopy images and schematics. B) Ionic strength‐responsive behavior of PAAm‐based microgels without nanogels, with homogeneous nanogel distribution and compartmentalized nanogels, as well as the compartment itself with *n* = 50 microgels measured for microgel area and *n* = 25 for compartment area. C) Microgel and compartment sizes of PAAm‐based NiM‐C in a series of different solvents sorted by decreasing polarity for *n* = 20 microgels.

First, the ionic strength‐responsive behavior of PNIPAAm‐ and AAm‐based NiM‐C containing uncharged nanogels was investigated in the range from 0 to 4000 mm. For this, the microgels without nanogels and NiM‐C with either homogeneous distribution of nanogels or compartmentalized nanogels were dispersed in salt solutions with varying concentrations of sodium chloride. The PNIPAAm NiM‐C exhibits ionic strength‐dependent deswelling known in the literature^[^ ^33^ ^]^ both in the nanogel compartment and in the surrounding microgel network (Figure S7B, Supporting Information). For all PAAm‐based microgels, the surrounding microgel networks behave similarly and independently from nanogel incorporation as seen in **Figure** **3**B. All three samples exhibit a slight decrease in size between 0 and 50 mm and stay at a constant size above an ionic strength of 50 mm. However, even after incorporation into PAAm‐based microgels, the PNIPAAm‐based nanogel compartments present with the ionic strength‐responsive behavior reported in the literature^[^ ^33^ ^]^ and seen for PNIPAAm NiM‐C (Figure 3B): Between 500 and 1000 mm salt concentration, the compartment collapses. The incorporation of nanogels does not affect the properties of the surrounding polymeric network and enables anisotropic shape change, where only part of the colloid changes in size. A similar behavior has been reported for Janus microgels with a PNIPAAm‐rich and PNIPAAm‐poor side.^[^ ^35^ ^]^ Although the PNIPAAm‐poor side exhibited less swelling than the PNIPAAm‐rich side, for PAAm NiM‐C the microgel network is completely non‐responsive and therefore, the anisotropic shape change is more pronounced.

Moreover, the compartment area in the PAAm‐based sample decreases to roughly 60% of its original size, while for the PNIPAAm NiM‐C the compartment shrinks to 40% of its original size (Figure S7B, Supporting Information). This shows that, while the compartments undergo a significant change in volume in both microgel species, either the non‐responsive PAAm network restricts the shrinking of the compartment or the responsive PNIPAAm network reinforces the shrinking of the compartment. These results reveal that the responsive behavior of the compartments is preserved, while not affecting the properties of the surrounding microgel network.

This anisotropic shape change effect can also be triggered by increasing the temperature above the VPTT of PNIPAAm to 40 °C (Figure S7C, Supporting Information). In addition, the influence of nanogel, AAm and BIS concentration on the deswelling of the PNIPAAm compartment in PAAm NiM‐C was investigated in Figure S7D,E (Supporting Information). As the nanogel concentration is increased, the compartment area tends to increase, while the change in deswelling does not seem to follow a trend. We found that changes in AAm concentration did not influence the deswelling, while a decrease in crosslinker density leads to a significant increase in deswelling as the microgel network decreases in stiffness.

In contrast to ionic strength and temperature, which can be used to control the compartment size, the addition of organic solvents results in a collapse of the surrounding PAAm network (Figure 3C; Figure S9, Supporting Information) because of the highly hydrophilic nature of PAAm. While the PAAm network collapses significantly, the area of the nanogel compartment changes only very slightly in all tested solvents because PNIPAAm is more hydrophobic than PAAm and can easily be dispersed in less polar solvents like iso‐propanol. When comparing water and ethylene glycol, the microgel area is very similar. The surrounding PAAm network is extremely swollen as the hydrophilic solvents are taken up into the network. In methanol, the PAAm network significantly decreases in size. By substituting the surrounding solvent to an even less polar solvent like ethanol or iso‐propanol, the PAAm matrix collapses further, which is visible in optical microscopy from the smaller diameter and darker color of the microgels. The slight decreases in PNIPAAm compartment area can partly be explained by the collapse of the surrounding network that, to some extent, also leads to compression of the internal compartment. In the optical microscopy images for the PAAm‐based microgels dispersed in organic solvents, the nanogel compartments seem to be expelled from the surrounding microgel network. However, Figure S10 (Supporting Information) shows that the NiM‐C are fully redispersable in water after being dispersed in organic solvents and regaining their original shape. Therefore, the nanogel compartment is not expelled from the microgel, but instead remains connected to the microgel and adopts a highly anisotropic shape due to two competing effects: the nanogel compartment being swollen in organic solvent and the collapsed microgel network compressing parts of the nanogel compartment. Lastly, the NiM‐C is also redispersable after drying (Figure S8, Supporting Information).

All in all, we showed that in PNIPAAm‐ and PAAm‐based NiM‐C the responsive behavior of the PNIPAAm‐based nanogel compartments stays intact, but is slightly promoted when integrated into the responsive PNIPAAm network. For PAAm NiM‐C, we investigated anisotropic shape change, where the size or volume of the separate compartments could be controlled independently from each other. The PNIPAAm compartment can be modulated by temperature and salt concentration, while a change in solvent polarity significantly influences the PAAm matrix. This effect is interesting for biomedical applications, for instance, where different compartments must be triggered separately for controlled drug release.

#### 2.2.2 Permeability Assays

**Figure 4:**
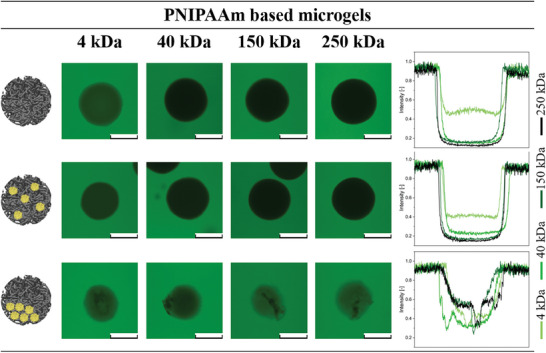
Permeability assay of pure PNIPAAm microgels, PNIPAAm NiM‐C with homogeneous nanogel distribution, and compartmentalized NiM‐C. The intensity of the fluorescent FITC‐dextrans (4 to 250 kDa average molecular weight) through the cross‐section of the microgels is plotted on the right. Scale bars represent 100 µm.

**Figure 5:**
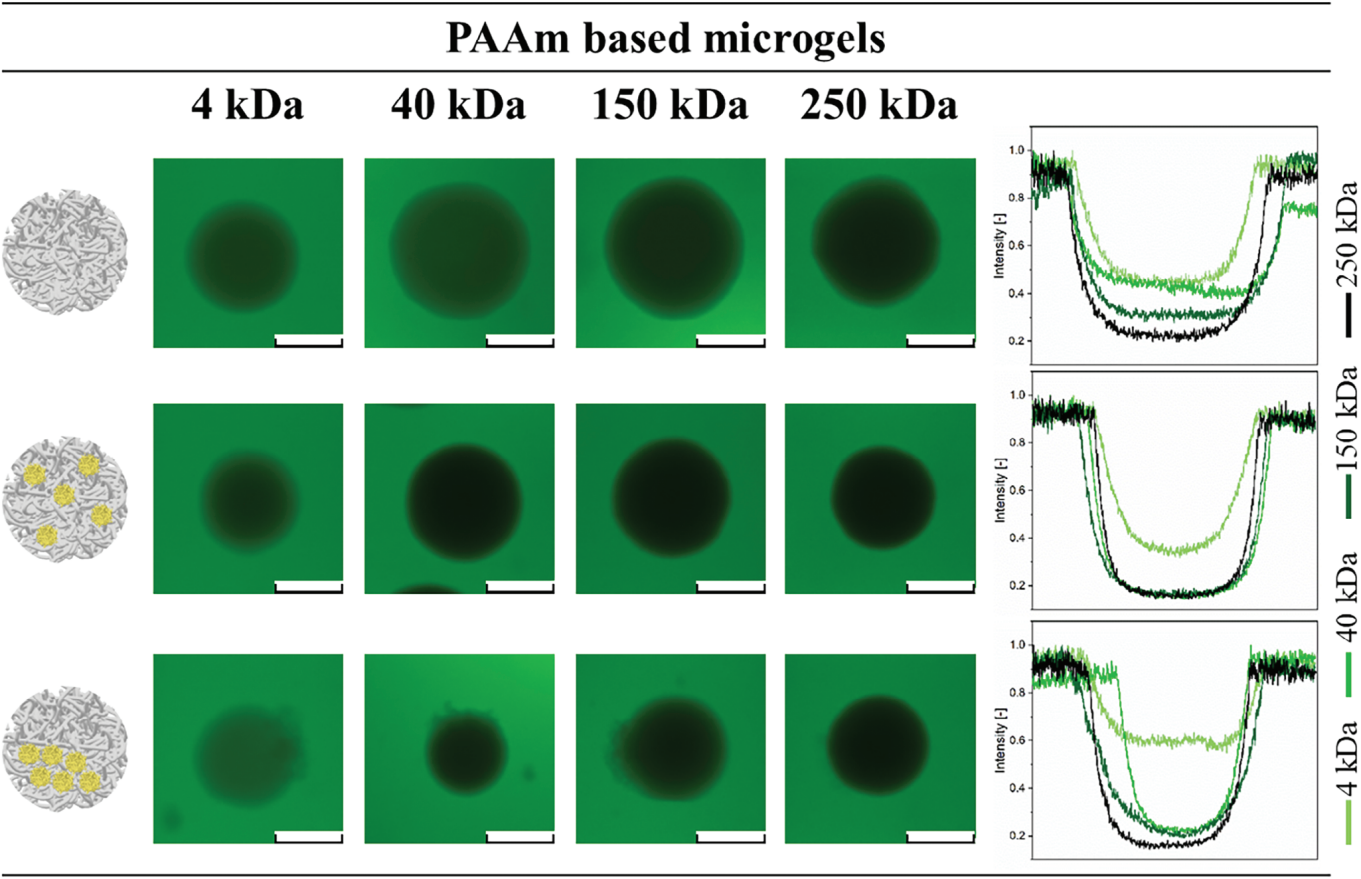
Permeability assay of pure PAAm microgels, PAAm NiM‐C with homogeneous nanogel distribution, and compartmentalized NiM‐C. The intensity of the fluorescent FITC‐dextrans (4 to 250 kDa average molecular weight) through the cross‐section of the microgels is plotted on the right. Scale bars represent 100 µm.

As compartmentalized microgels find application in drug delivery, investigating the influence of nanogel incorporation and compartmentalization on particle permeability is crucial. For this purpose, permeability assays were performed for both the PNIPAAm‐ (**Figure** **4**) and PAAm‐based microgels (**Figure** **5**) containing no nanogels, homogeneously distributed nanogels, and compartmentalized nanogels. For these experiments, microgel or NiM‐C dispersions were mixed with aqueous fluorescein isothiocyanate dextran (FITC‐dextran) solutions of different molecular weights (4, 40, 150, and 250 kDa) and subsequently visualized in CLSM.

The pure PNIPAAm microgels without nanogels and the PNIPAAm NiM‐C with homogeneously distributed nanogels are only penetrated by the smallest 4 kDa FITC‐dextran, while the higher molecular weight dextrans cannot diffuse inside. For the second sample with homogeneous nanogel distribution, the fluorescence intensity of the 4 kDa FITC‐dextran is slightly lower, indicating that the nanogels decrease the permeability by creating a denser network. 4 kDa FITC‐dextran molecules have hydrodynamic diameters of ≈1.3 nm,^[^ ^36^ ^]^ indicating that the PNIPAAm microgels exhibit a similar pore size. Nonetheless, this permeability assay only gives a rough estimate of the pore sizes as permeability is not only influenced by particle size but also other factors like interactions between microgel and dye. Moreover, the intensity profiles show a sharp drop in intensity at the microgel‐water interface and overall constant intensity inside the microgels, which suggests a sharply defined surface and homogeneous interior.

Interestingly, for the compartmentalized PNIPAAm NiM‐C, the fluorescence intensity is higher throughout the microgel network for all FITC‐dextrans and similar among the different molecular weights, while the diffusion of the dextrans into the compartments is very limited. As even 250 kDa can penetrate the microgel network in the compartmentalized sample, the pore size is at least 11.4 nm,^[^ ^36^ ^]^ which corresponds to a pore size approximately ten times higher than for the homogeneous samples. This increase in permeability in compartmentalized samples is reproducible (Figure S11, Supporting Information) and also observable in PNIPAAm‐based microgels synthesized at high ionic strengths without nanogels (see Figure S12, Supporting Information). This phenomenon can be explained by the ionic strength‐responsiveness of PNIPAAm: The UV irradiation initiates the polymerization of NIPAAm that leads to the formation of PNIPAAm chains, which exhibit ionic strength‐responsive behavior and precipitate at high salt concentrations present in the droplets. The precipitation occurring during the polymerization leads to a more porous network structure even in samples that do not contain nanogels (Figure S12, Supporting Information). This hypothesis is supported by the fuzzy surface structure of PNIPAAm‐based NiM‐C as seen in brightfield images (Figure 2). In the confocal images, the compartmentalized NiM‐C is slightly smaller and less spherical than the other two samples. Small deviations in microgel sizes between samples can be a result of the precipitation of the nanogels near the cross junction, which can cause a change in droplet size. A more anisotropic appearance of the compartmentalized NiM‐C can be explained by the swelling of the nanogel compartment inside the microgel after purification. Salt has been used to produce porous hydrogels before. For instance, porous poly(ethylene glycol)^[^ ^37^ ^]^‐ or hyaluronic acid‐based hydrogels^[^ ^38^ ^]^ can be prepared by adding salt crystals to the precursor solution and leaching the salt out after polymerization. However, this method has not been applied to microgels and requires two steps, while the porosity of the herein‐presented PNIPAAm microgels is increased in only one step.

In contrast to the PNIPAAm‐based samples, most PAAm microgels exhibit a gradient‐like decrease in fluorescence intensity from the microgel surface toward the microgel core. This indicates a loosely crosslinked periphery and less homogeneous network interior. The pure PAAm microgels take up a slightly higher amount of the 4 kDa FITC‐dextran than the pure PNIPAAm microgels. While their PNIPAAm‐based counterparts do not take up any of the larger FITC‐dextrans, the uptake of 40 kDa FITC‐dextrans, corresponding to 4.5 nm,^[^ ^36^ ^]^ is significantly improved and even the 150 kDa dextrans can partly diffuse into the pure PAAm microgels. Therefore, the PAAm‐based microgels are more permeable overall, possibly because of more swelling.

The NiM‐C that contains homogeneously distributed nanogels exhibits considerably denser and less porous networks, as only the 4 kDa FITC‐dextran molecules are able to diffuse into the NiM‐C, similar to the comparable PNIPAAm sample. In this case, it is clear that the nanogels lead to a denser and less permeable microgel network. The last sample, the compartmentalized PAAm NiM‐C, behaves very similar to the sample with the homogeneous nanogel distribution. Here, the compartments are similar in permeability to the surrounding network, and the smoother surface structure of the compartmentalized NiM‐C is also similar to the pure PAAm microgels in brightfield microscopy. This observation confirms the aforementioned hypothesis that the formed PNIPAAm polymer chains start precipitating during polymerization. As PAAm is non‐responsive, this effect does not occur and the microgels synthesized with and without the presence of salt are similar in texture and permeability.

In summary, the PAAm‐based microgels are permeable for small molecules, while the incorporation of nanogels inside NiM‐C decreases the permeability. Consequently, the permeability is adjustable and can be tailored for specific applications. Additionally, we found that ionic strength‐responsive materials like PNIPAAm will undergo phase separation during polymerization in the presence of salt resulting in more porous network structures. Overall, the addition of nanogels as well as the presence of salt during the polymerization of ionic strength‐responsive polymers are valuable tools for tuning the pore size and permeability of synthesized microgels and NiM‐C.

### 2.3 Selective Uptake of Curcumin

**Figure 6:**
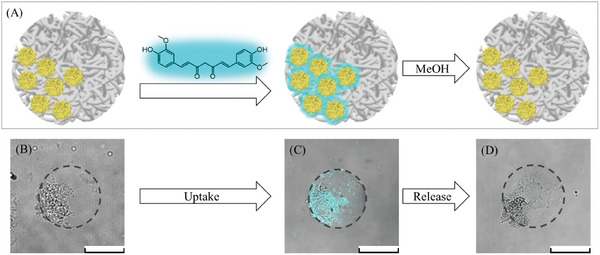
A) Schematic representation of PAAm NiM‐C with compartments containing unlabelled PNIPAAm nanogels used for uptake of the hydrophobic model drug curcumin (cyan) and its subsequent release in methanol. CLSM images show a NiM‐C B) before uptake, C) after uptake D) and after release. Scale bars represent 100 µm.

The NiM‐C presented in this work are model particles used for the development of the ionic strength‐induced compartmentalization method. To prove that this technique can be used for the production of microgels with distinct compartments that enable selective uptake and subsequent triggered release, exemplary experiments were performed (**Figure** **6**A). For this, curcumin was chosen as the model drug as it is hydrophobic^[^ ^39^ ^]^ and therefore expected to accumulate in the more hydrophobic PNIPAAm compartments. In addition, it exhibits fluorescence that can be imaged using CLSM. The NiM‐C used in this experiment are based on PAAm and contain uncharged nanogels that were synthesized without the fluorescent comonomer RB. This ensures that no fluorescence from the nanogels is detected during measurements and only the model drug curcumin is visualized.

Figure 6C depicts the results of the uptake experiment. Curcumin is shown in cyan and selectively accumulates in the more hydrophobic PNIPAAm compartments. Additionally, a z‐stack was performed (Figure S13, Supporting Information) to confirm that curcumin is present throughout the whole compartment. The compartmentalization is crucial for the uptake to work because a homogeneous distribution of nanogels does not yield a hydrophobic pocket in the microgel and therefore cannot be used for the uptake of curcumin (Figure S14, Supporting Information). After the uptake, methanol was added to the aqueous NiM‐C dispersion to release the curcumin (Figure S14, Supporting Information). Figure 6D confirms the successful release of the model drug after redispersion of the NiM‐C in water.

These exemplary experiments prove that the presented method allows for the production of microgels with compartments that exhibit vastly different properties and can be employed for the selective uptake and release of drugs.

## 3 Conclusion

In the presented work, the ionic strength‐induced compartmentalization technique in droplet‐based microfluidics was developed and optimized for uncharged, negatively and positively charged nanogels in PNIPAAm‐ and PAAm‐based nanogel‐in‐microgel colloids (NiM‐C). We found that combining both charged nanogel species led to their co‐precipitation due to the entanglement of loose chain ends. For all investigated combinations, the degree of compartmentalization is precisely controllable by adjusting the salt concentration. Additionally, microgels with iron oxide nanoparticle compartments were produced to further show the range of this method.

Ionic strength‐ and temperature‐dependent measurements of PNIPAAm and PAAm NiM‐C confirmed that the properties of the incorporated nanogels and the surrounding microgel remained intact. For compartmentalized PAAm NiM‐C in particular, the combination of responsive compartments embedded in non‐responsive microgels resulted in anisotropic shape change behavior: The PNIPAAm‐compartment can be triggered with changes in temperature or salt concentration, while the PAAm matrix collapses in organic solvents. Further, permeability assays showed that nanogel incorporation slightly decreased NiM‐C permeability. In contrast, the presence of salt during the polymerization of ionic strength‐responsive monomers like NIPAAm yielded more porous microgels as a result of generated polymer chains precipitating during polymerization. Lastly, in an exemplary uptake experiment the hydrophobic model drug curcumin was loaded into PNIPAAm‐rich compartments in PAAm NiM‐C confirming the presented method can be used for the fabrication of compartmentalized microgels for highly selective uptake and subsequent release.

In conclusion, we presented a novel method for the production of compartmentalized microgels that enables facile and precise control over the degree of compartmentalization and is applicable to a wide variety of systems. The method can be used to produce microgels that exhibit anisotropic shape change and can selectively take up smaller molecules. Therefore, the presented approach paves the way toward advanced microgel morphologies for biomedical applications.

## 4 Experimental Section

### Materials

*N*‐isopropylacrylamide (NIPAAm, 97%, recrystallized from n‐hexane), methacrylic acid (MAAc, 99%, filtered in a column with silica), *N,N′*‐methylenebis(acrylamide) (BIS, 99%), 2,2′‐azobis(2‐methylpropionamidine)dihydrochloride (AMPA, 97%), lithium‐phenyl‐2,4,6‐trimethylbenzoylphosphinate (LAP, ≥95%), Span 80 (nonionic surfactant), dimethyl sulfoxide (DMSO, ≥99.5%), hydrochloric acid (1 m, 0.1 m), sodium dodecyl sulfate (SDS, ≥99.0%), FITC‐dextrans, 3‐(cyclohexylamino)‐1‐propanesulfonic acid (CAPS, ≥99.0%), monopotassium phosphate (≥99.0%), succinic acid (≥99.0%), formic acid (≥96 %), sodium phosphate dibasic (≥99.0 %) and trishydroxymethylaminomethane (TRIS, ≥99.9%) were obtained from Merck (Sigma Aldrich). *N*‐(3‐aminopropyl)methacrylamide hydrochloride (APMH, >98%) was purchased from abcr. Acryloxyethyl thiocarbamoyl Rhodamine B (RB) was provided by Polysciences, and Cyanine5‐NHS ester (Cy5‐NHS) was obtained from Lumiprobe. 2,2′‐azobis(*N*‐(2‐carboxyethyl)−2‐methylpropionamidine)tetrahydrate (ACMA, min. 95.0%) was bought from WakoChemicals. Iso‐propanol (≥99.6 %), sodium hydroxide solution (1 m), and hexane (≥98.5%) were obtained from VWR. Sodium chloride (≥99.8%) was bought from Carl Roth. 1,4‐dioxane (≥99.8%) and potassium hydroxide (min 85.0%) were purchased from ChemSolute. PDMS silicone elastomer kit (SYLGARD 184) was purchased from Downsil. A mixture of the uncharged surfactant FluoSurf with HFE (hydrofluoroether) oil (FluoSurf 2 w/w% in HFE 7500) was obtained from Emulseo. HPLC‐grade water was obtained from VWR. AccuGENE 10X PBS was purchased from Lonza Bioscience. EMG 700 metal nanoparticles were used as received from FerroTec.

### Synthesis of PNIPAAm‐Based Nanogels

The uncharged PNIPAAm‐based nanogels were synthesized by precipitation polymerization. NIPAAm (2263.20 mg, 20 mmol, 100 mol%), BIS (61.67 mg, 0.4 mmol, 2 mol%), and SDS (1.30 mg, 0.005 mmol, 0.15 mol%) were dissolved in 196.2 mL HPLC‐grade water and transferred into a double‐wall glass reactor. The reaction mixture was heated to 70 °C and purged with nitrogen for 45 min while continuously stirring. Separately, ACMA (124.34 mg, 0.3 mmol, 1.5 mol%) was dissolved in 2 mL HPLC water and flushed with nitrogen for 45 min. 800 µL 0.01 m HCl was added to the monomer solution. The reaction was started by injection of the ACMA solution. After 3 min, RB (6.7 mg, 0.01 mmol, 0.05 mol% in 1 mL HPLC‐grade water) was added to the reaction mixture, and the polymerization was carried out in the dark for 4 h under a nitrogen atmosphere and stirring. The product dispersion was purified by automated tangential‐flow diafiltration against demineralized water (min. 5 L) using PES membranes (molecular weight cutoff: 30 kDa, membrane area: 0.6 m^2^).

The positively and negatively charged nanogels were synthesized according to Mathews and Pieper et al.^[^ ^30^ ^]^

### DLS and ELS Measurements

For the DLS and ELS measurements, a ZetaSizer Ultra from Malvern Panalytics was used with cells of the type DTS0012 or DTS1070 and analyzed with the ZS Xplorer software. To prepare the DLS samples, 100 µL of the nanogel dispersion was filled into a snap cap vial and mixed with 2 mL of a buffer or solution. Cuvettes were filled to ≈1 cm with a syringe through a 1.2 µm PET syringe filter. All measurements were performed at 22.5 °C and 90° or 174.7° for side and backscattering, respectively. Each data point was averaged over three measurements.

### Fabrication of Droplet‐Based Microfluidic Devices

A blueprint of the microfluidic device was designed with the AutoCAD software (Autodesk, USA) and printed on a dark‐field photomask (25 000 dpi). The pattern was transferred onto an SU‐8 epoxy‐based photoresist attached to a silicon wafer via soft photolithography to obtain a master mold according to Bulut and Günther et al.^[^ ^36^ ^]^ For replica molding, polydimethylsiloxane (PDMS) was mixed with a curing agent (Silgard 184 elastomer kit, Dow Corning USA, ratio of PDMS:crosslinker 10:1). The mixture was degassed and transferred into the master mold, which was cured at 60 °C overnight. Subsequently, the PDMS chip was cut from the master mold. Three inlets (one for the oil phase, two for the water phases) and one outlet were punched in the microfluidic channel with a biopsy puncher (MicrotoNano, Haarlem Netherlands, diameter: 0.75 mm). The PDMS chip was washed three times with water and iso‐propanol each. The PDMS chip and a glass slide were fused together through oxygen plasma bonding (30 mL min^−1^, 100 W, 40 s) and reheating (120 °C, 5 min). Lastly, the microchannels were flushed with Aquapel (Pittsburgh Glass Works USA) to ensure a hydrophobic channel surface. To remove excess Aquapel solution, the microfluidic channels were flushed with air using an empty syringe. All microfluidic devices had channels with a diameter of 80 µm.

### Droplet‐Based Microfluidic Synthesis

Two syringe pumps (PHD Ultra, Harvard Apparatus, Holiston USA) were connected to a microfluidic PDMS device with fine‐bore polyethylene tubing (inner diameter: 0.40 mm, outer diameter: 1.10 mm). Droplet formation was observed with an inverted microscope (Motic AE2000, TED PELLA Inc., Redding CA) and recorded with a camera (Flea3, Point Grey, Richmond CA). For the first aqueous phase, NIPAAm (98.4 mg, 0.87 mmol, 100 mol%) or AAm (61.8 mg, 0.87 mmol, 100 mol%) as well as BIS (3.4 mg, 0.02 mmol, 2.5 mol%) and LAP (10 mg, 0.03 mmol, 1 wt.%) were dissolved in 0.5 mL of a nanogel dispersion of a certain concentration in HPLC‐grade water (or pure HPLC‐grade water for the samples that do not contain nanogels). For the second aqueous phase, sodium chloride was dissolved in HPLC‐grade water at the desired concentration (or pure HPLC‐grade water for the samples without nanogels or with homogeneous nanogel distribution). For the continuous oil phase, fluorocarbon oil (HFE‐7500) with 2 wt.% FluoSurf surfactant was employed. FluoSurf is a neutral surfactant that was chosen because it does not interact with the charges of the nanogels. The volume flow rates were set to 150 µL h^−1^ for both water phases and to 600 µL h^−1^ for the oil phase. The obtained water‐in‐oil emulsion was placed under UV light (3.6 W, 365 nm) for 10 s to initiate the polymerization. For the purification, the samples were washed three times with HFE‐7500 oil by adding the solvent, shaking, and removing the excess oil after sedimentation of the microgels. This was repeated twice with a mixture of hexane and 1% Span80, twice with pure hexane, twice with 1,4‐dioxane, three times with iso‐propanol, and three times with HPLC‐grade water. For the washing steps with dioxane, iso‐propanol, and water, the samples were centrifuged at 8000 rpm for 15 to 30 s to accelerate the sedimentation of the microgels.

For the production of microgels containing metal nanoparticle compartments, NIPAAm (98.4 mg, 0.87 mmol, 100 mol%) or AAm (61.8 mg, 0.87 mmol, 100 mol%) as well as BIS (3.4 mg, 0.02 mmol, 2.5 mol%) and LAP (10 mg, 0.03 mmol, 1 wt.%) were dissolved in 0.5 mL HPLC‐grade water before adding 2 µL of EMG 700. For the second aqueous phase, an X10 PBS buffer was used. During the reaction, ≈7 cm of the microfluidic tubing was irradiated continuously to ensure complete irradiation of every single droplet due to the high absorption exhibited by the nanoparticles. The rest of the synthesis and purification were done in the same manner as for the production of the NiM‐C.

### Confocal Laser Scanning Microscopy

For confocal laser scanning microscopy (CLSM), the Leica TCS SP8 confocal microscope (Leica, Wetzlar, Germany) was used. Rhodamine B was excited with a DPSS laser at a wavelength of 561 nm, Cyanine5‐NHS ester was excited with a HeNe laser at 633 nm and curcumin was excited with a diode laser at 405 nm (all at 1–5% intensity). For fluorescence images, HyD2 (≈ 300% gain; pinhole 1.00) and for brightfield images, PMT detectors were used. RB was detected between 575 and 620 nm, while Cy5 was detected in the range from 650 to 750 nm and curcumin from 520 to 640 nm. For permeability studies, microgel dispersions were mixed with 1 mg mL^−1^ FITC‐dextran solutions (4, 40, 150, and 250 kDa). FITC‐dextrans were excited using an Argon laser at 488 nm. Fluorescence was detected in the range of 500 to 550 nm. The intensity profiles were generated with LAS X software, and normalized and plotted in Origin. For PAAm‐based samples, the visibility of the interface between microgel and solution was enhanced either by shifting the z‐plane so the microgels are slightly out of focus, or by adjusting the images in contrast, which was used to add a dotted line around the microgels in confocal images.

### Image Analysis for Responsive Behavior

Temperature‐dependent measurements were performed on a heating stage (Tokai‐Hit ThermoPlate). For ionic strength‐dependent measurements, 10 µL of a microgel dispersion was mixed with 10 µL of a salt solution with double the desired concentration. Images for investigation of thermo‐ and ionic strength‐responsiveness were evaluated using ImageJ software. For the determination of the deswelling of the microgels when increasing the temperature, the equation 
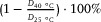
 was used (with 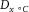 being the diameter determined via image analysis at temperature *x* °C). For instance, if the microgel would shrink from 100 µm at 25 °C to 50 µm at 45 °C, the deswelling would amount to 50 %. The diagrams were plotted in Origin. For all graphs that include an average and standard deviation, the investigated sample size *n* is given in the Figure description.

### Uptake Experiment

Fifty microliter of a concentrated NiM‐C dispersion was transferred into a 1.5 mL Eppendorf tube. 0.5 mL HPLC‐grade water and 0.5 mL of a 3 mg mL^−1^ curcumin in methanol solution were added and the solution was mixed. The sample was shaken for 24 h at room temperature in the dark. The NiM‐C were purified by washing with pure methanol once and water three times. In between washes, the dispersion was centrifuged at 7000 rpm for 15 s to accelerate the sedimentation of the particles.

## Conflict of Interest

The authors declare no conflict of interest.

## Author Contributions

M.I.P. and H.F.M. developed the concept and planned the experiments with the support of A.P. M.I.P. performed the synthetic experiments and carried out the CLSM experiments. M.I.P. recorded and analyzed microscopy images. M.I.P. designed the graphics. M.I.P. wrote the manuscript with the help of H.F.M. and A.P. All authors reviewed this manuscript and approved the final version.

## Supporting information



Supporting Information

## Data Availability

The data that support the findings of this study are available from the corresponding author upon reasonable request.
