# Supplementary material for: Ionic Strength‐Induced Compartmentalization for Nanogel‐in‐Microgel Colloids
Source: Small. 2025 Jan 15;21(9):2410221. doi: 10.1002/smll.202410221 (PMC11878252; doi:10.1002/smll.202410221)
Supplement: Supplementary file 1 — Supporting Information [file SMLL-21-2410221-s001.docx]

Supporting Information

Ionic Strength-Induced Compartmentalization for Nanogel-in-Microgel Colloids

Maria I. Pieper, Hannah F. Mathews, Andrij Pich*


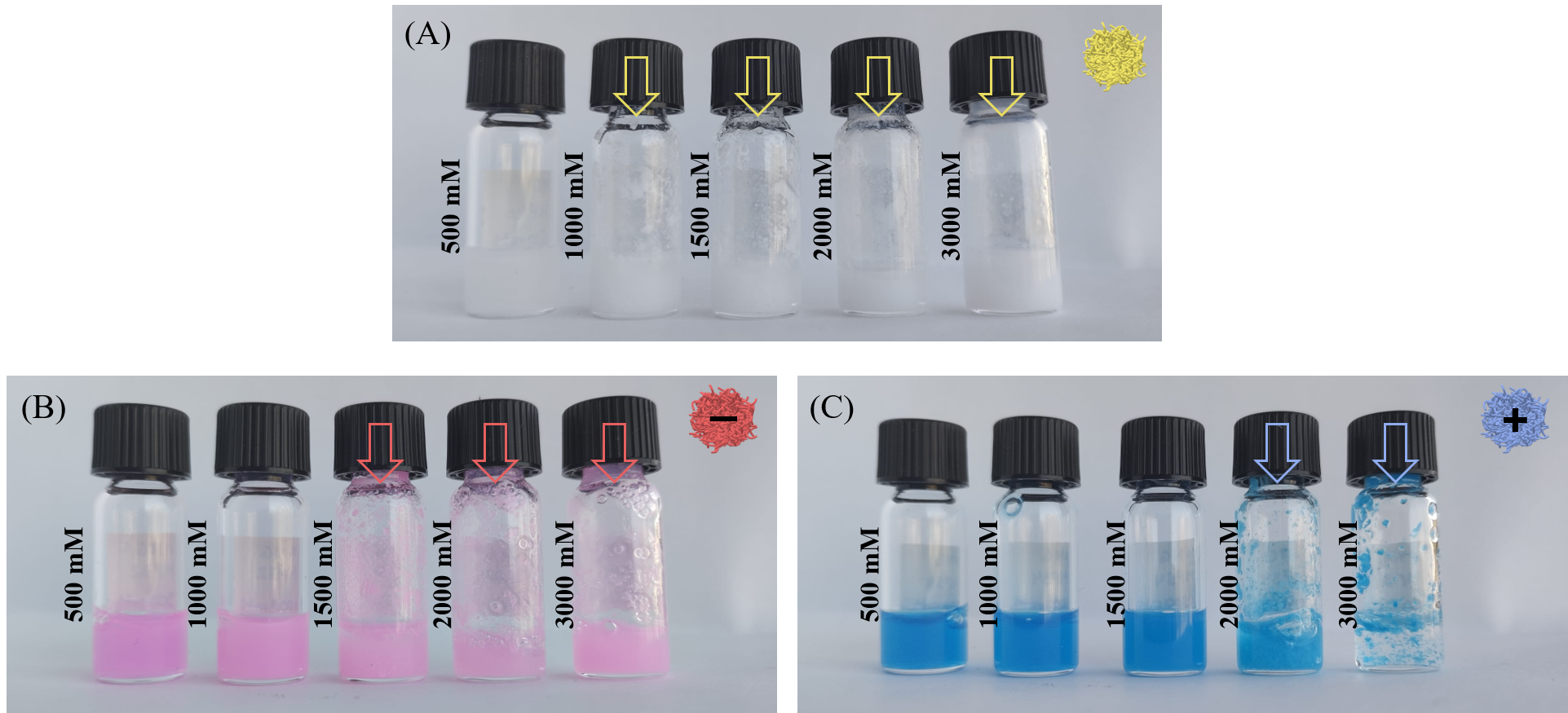


**Figure S1.** Photographic images of (A) uncharged, (B) negatively charged and (C) positively charged nanogel dispersions at salt concentrations of 500, 1000, 1500, 2000 and 3000 mM. The downward arrows denote the salt concentration at which the nanogels collapse and the dispersion separates into two distinct phases.

**Figure S1** demonstrates that the uncharged nanogels precipitate at 1000 mM, the negatively charged nanogels at 1500 mM and the positively charged nanogels at 2000 mM. In the PNIPAAm-based microgels, the nanogels precipitate at approximately 300, 1250 and 1500 mM, respectively. Apparently, the components dissolved in the nanogel dispersion for the microfluidic reactions, like the monomer, crosslinker and initiator, lead to a collapse of the nanogels at lower salt concentrations. The visual analysis of the nanogel dispersions at different ionic strengths shows if the nanogels can be used for the herein presented ionic strength-induced compartmentalization. However, as the solutions in the microfluidic setup are not pure nanogel dispersions, other components and interactions need to be considered. Therefore, the optimization of the ionic strength used in microfluidic synthesis must be performed for every system.

To characterize the nanogel samples, especially in regard to ionic strength-responsive behavior, DLS and ELS measurements were performed analogous to previous studies.^[1]^ For this, buffers of different ionic strengths were prepared by dissolving sodium chloride in HPLC-grade water at the desired concentrations. Additionally, buffers of different pH values were prepared with constant ionic strengths of 10 mM, so that the influence of the ionic strength on the nanogel size and charge is negligible. For the buffer at pH 3.5, 25.88 mL of 0.1 M formic acid and 9.65 mL of 0.1 M KOH were mixed with 64.47 mL water. For pH 5, 38.12 mL 0.02 M succinic acid and 16.80 mL 0.05 M KOH were mixed with 45.08 mL water. For pH 7, 13.96 mL 0.02 M potassium phosphate monobasic and 24.04 mL 0.01 M sodium phosphate dibasic were mixed with 62.00 mL water. For pH 8.9, 55.22 mL 0.1 M TRIS and 10.00 mL 0.1 M HCl were mixed with 34.78 mL water. For pH 11, 0.99 mL 1 M NaOH, 0.0471 mL 1 M NaCl and 0.29 g of CAPS were mixed with 98.54 mL water.


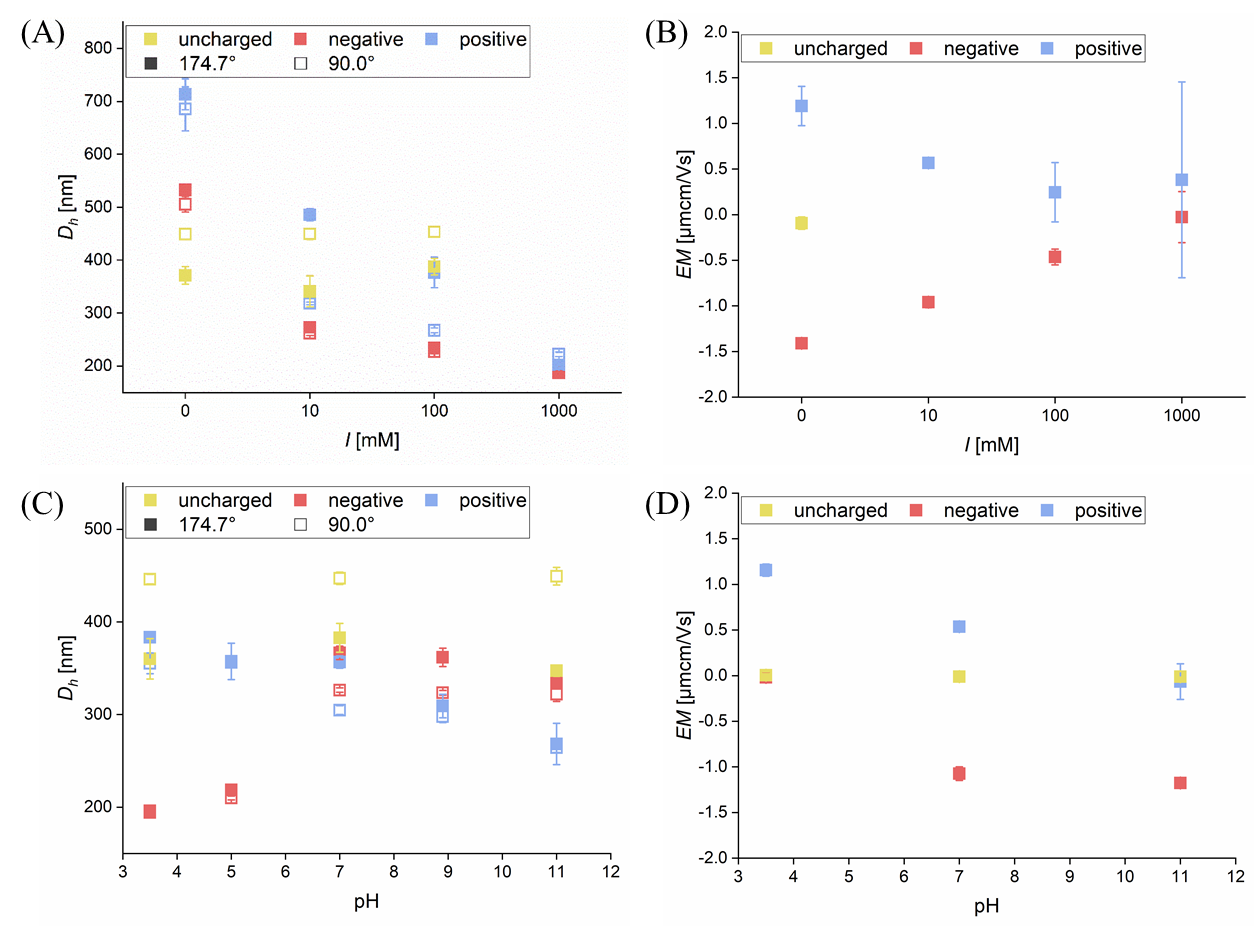


**Figure S2.** DLS (A) and ELS results (B) in dependence on ionic strength. DLS (C) and ELS (D) results in dependence on pH value. All measurements were performed at 22.5 °C for uncharged (yellow), negatively charged (red) and positively charged (blue) nanogels. For DLS measurements, side scattering was performed at an angle of 90.0° and is shown as empty square, while back scattering was performed at 174.7° and is shown as filled square. The size of the uncharged nanogels could not be measured at 1000 mM because the sample agglomerated.

The nanogels behaved as previously reported.^[1]^ The ionic strength-dependent DLS measurements (**Figure S2 (A)**) show that while the uncharged nanogels retain their original size up to an ionic strength of 100 mM, the charged nanogels exhibit a continuous decrease in size as a result of the charges getting screened by the electrolytes. At 1000 mM, the charged nanogels decrease only slightly in size compared to 100 mM, while the uncharged sample exhibited aggregation and therefore could not be reasonably measured. The ELS measurements (**Figure S2 (B)**) confirm the uncharged, negatively and positively charged character of the respective nanogels. At increasing salt concentrations, the charges are screened and the electrophoretic mobility approaches a value of 0.

In the pH-dependent DLS measurements (**Figure S2 (C)**), the uncharged nanogels stay at a constant size, confirming their non-responsive behaviour regarding changes in pH value. In the case of negatively charged nanogels, the nanogels are swollen at pH 7 and above, since the repulsive forces between the charges and the increased osmotic pressure inside the nanogels lead to a swelling at high pH values. At pH values of 5 and 3.5, the nanogels collapse because the charges are neutralized in acidic medium. The positively charged nanogels exhibit the opposite behaviour: The nanogels are charged and swollen at low pH and decrease in size due to the neutralization of the charges towards high pH values. This behaviour is additionally confirmed by pH-dependent ELS measurements (**Figure S2 (D)**).

The amounts of chemicals used for the precipitation polymerization of uncharged PNIPAAm‑based nanogels are shown in **Table S1**. NIPAAm, BIS and SDS were dissolved in 196.2 mL HPLC-grade water before adding 800 µL 0.01 M hydrochloric acid. ACMA was dissolved in 2 mL HPLC-grade water. Both solutions were degassed with nitrogen for 45 minutes. RB was dissolved in 1 mL HPLC-grade water and added to the reaction 3 minutes after initiation.

**Table S1.** Exact masses and amounts of chemicals used for precipitation polymerization of uncharged PNIPAAm nanogels.

|  | Molecular weight *M_w_* [g mol^-1^] | Mass *m* [mg] | Amount *n* [mmol] | mol% |
| --- | --- | --- | --- | --- |
| NIPAAm | 113.16 | 2263.2 | 20.00 | 100.00 |
| BIS | 154.17 | 61.7 | 0.40 | 2.00 |
| SDS | 288.38 | 8.7 | 0.03 | 0.15 |
| ACMA | 342.39 | 12.4 | 0.30 | 1.50 |
| RB | 666.20 | 6.7 | 0.01 | 0.05 |

**Table S2** and **Table S3** list the amounts of NIPAAm or AAm, BIS and LAP used in droplet-based microfluidic synthesis. The chemicals were dissolved in 0.5 mL of a nanogel dispersion for the reaction. The total concentration is halved after droplet formation, which is why the desired salt concentration in the droplets is doubled in the initial solution used in the microfluidic synthesis.

**Table S2.** Exact masses and amounts of chemicals used for PNIPAAm-based microgels and NiM-C in droplet-based microfluidics per 0.5 mL solution or dispersion.

|  | Molecular weight *M_w_* [g mol^-1^] | Mass *m* [mg] | Amount *n* [mmol] | mol% |
| --- | --- | --- | --- | --- |
| NIPAAm | 113.16 | 98.4 | 0.87 | 100.00 |
| BIS | 154.17 | 3.4 | 0.02 | 2.50 |
| LAP | 294.21 | 10.0 | 0.03 | 3.91 |


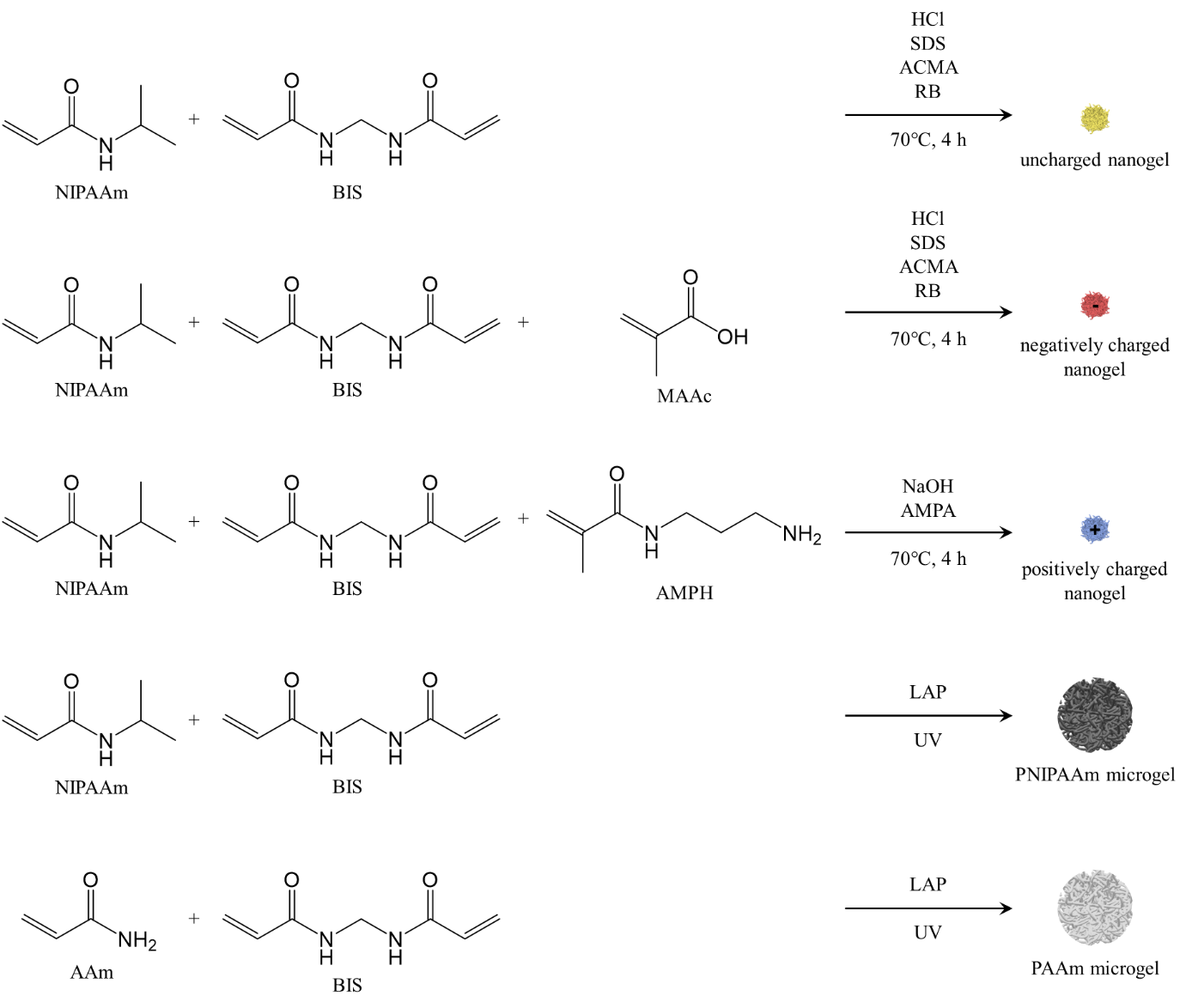
**Table S3.** Exact masses and amounts of chemicals used for PAAm-based microgels and NiM‑C in droplet-based microfluidics per 0.5 mL solution or dispersion.

|  | Molecular weight *M_w_* [g mol^-1^] | Mass *m* [mg] | Amount *n* [mmol] | mol% |
| --- | --- | --- | --- | --- |
| AAm | 71.08 | 61.8 | 0.87 | 100.00 |
| BIS | 154.17 | 3.4 | 0.02 | 2.50 |
| LAP | 294.21 | 10.0 | 0.03 | 3.91 |

The simplified reactions from **Table S1**, **Table S2** and **Table S3** for the fabrication of charged and uncharged nanogels as well as PNIPAAm- and PAAm-based microgels in droplet-based microfluidics are shown in **Figure S3**.

**Figure S3.** Simplified overview over precipitation polymerization reactions used for the synthesis of the nanogels and photo-initiated polymerizations employed in droplet-based microfluidics for the fabrication of PNIPAAm- and PAAm-based microgels.

**Figure S4.** Schematic representation of droplet-based microfluidics setup for the exemplary reaction for PAAm-based NiM‑C with uncharged nanogel compartments. (A) The two aqueous phases containing nanogels, monomer, crosslinker and photo initiator, and salt meet at the cross junction. (B) In the mixing section, the droplets are mixed as the laminar flow is changed to turbulent flow. (C) In the outlet, the water-in-oil droplets are collected subsequently irradiated with UV light. The scale bars represent 500 µm.


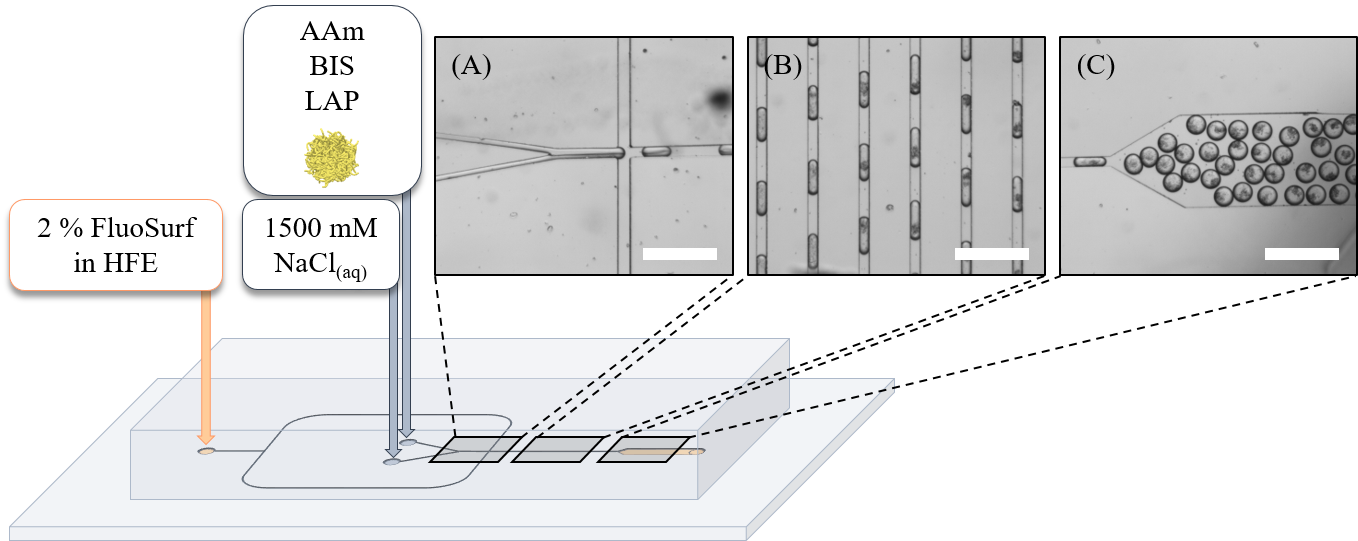


For the exemplary reaction for the production of PAAm NiM-C with uncharged nanogel compartments, a dark line is visible at the water-water interface before the cross junction (**Figure S4** **(A)**). This results from the locally very high salt concentration at the interface, which leads to nanogel precipitation. In the mixing section (**Figure S4 (B)**), the droplets are mixed as the laminar flow is changed to turbulent flow. When the salt concentration is homogenized throughout the droplets, the nanogels form more and more phase-separated compartments. This becomes more pronounced the further the droplets flow through the mixing section (from left to right). In the outlet (**Figure S4** **(C)**), the nanogels have formed distinct compartments, which are subsequently immobilized by UV irradiation. Observing the droplet formation this way allows for instant feedback over compartmentalization: If the clogging at the cross junction leads to jetting and polydisperse droplets, the ionic strength needs to be reduced; if no compartments form in the mixing section and outlet, the ionic strength needs to be increased. This microfluidic approach allows for facile and rapid adjustment of the ionic strength as only the salt solution in one syringe must be exchanged. Even though the ionic strength must be optimized for each system, this way the optimization can be done rapidly (especially after pre-experiments like in **Figure S1**).


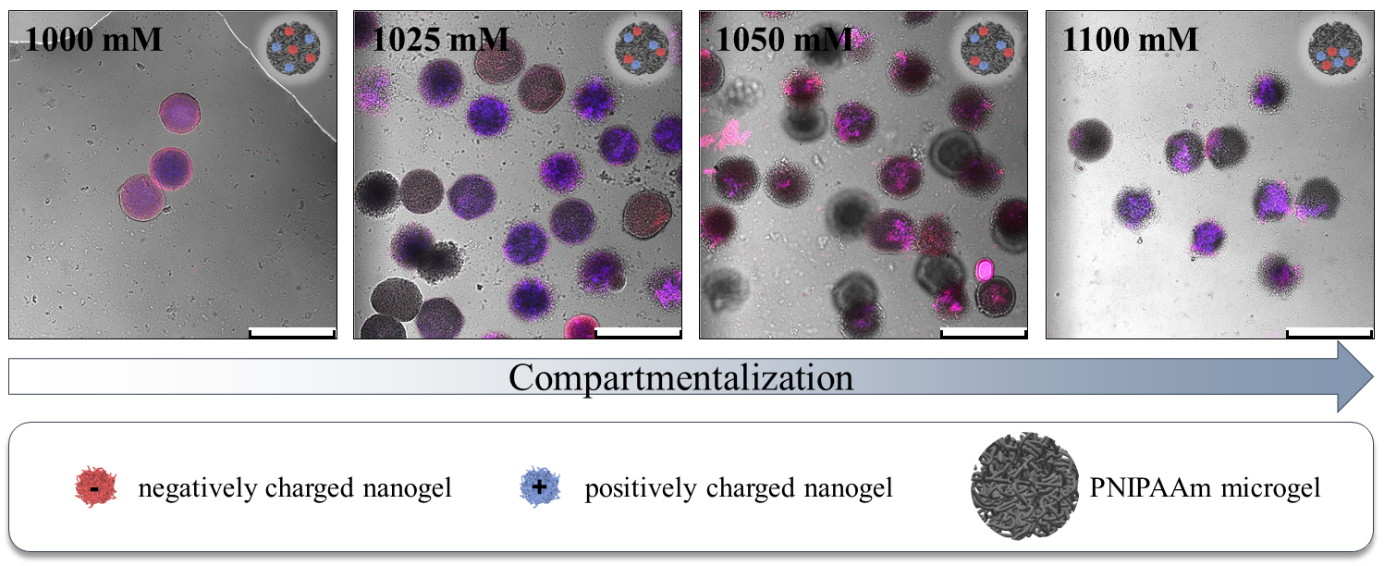
**Figure S5.** CLSM images of both positively (blue) and negatively charged nanogels (red) in PNIPAAm microgels at different ionic strengths. The given ionic strengths were present in the droplet after mixing of both aqueous phases. Compartmentalization is more pronounced with increasing ionic strength. Scale bars represent 300 µm.

Exemplary, CLSM images of PNIPAAm-based NiM-C containing a combination of negatively and positively charged nanogels with varying ionic strengths during synthesis are shown in **Figure S5**. Similar to the uncharged NiM-C (**Figure 1**) the nanogel distribution can precisely be controlled by varying the ionic strength. Here, a small difference in ionic strength of 25 mM makes a remarkable difference in nanogel distribution. The degree of compartmentalization is quantified in **Table S4**. At 1000 mM, all microgels contain homogeneously distributed nanogels. At 1025 mM, around 33 % of microgels contain compartments, which increases to 88 % at 1050 mM. At the highest ionic strength, 1100 mM, all microgels are compartmentalized.

**Table S4.** Fraction of compartmentalized microgels for samples shown in **Figure 1**, **Figure 2** and **Figure S5**. For each sample a minimum of *n* = 20 microgels were evaluated.

| **PNIPAAm-based microgels** | | |
| --- | --- | --- |
| **nanogel type** | ***I* [mM]** | **Fraction of compartmentalized microgels [%]** |
| uncharged | 100 | 8 |
|  | 200 | 50 |
|  | 300 | 100 |
|  | 500 | 100 |
| negatively charged | 1250 | 80 |
| positively charged | 1500 | 47 |
| negatively + positively charged | 1000 | 0 |
|  | 1025 | 33 |
|  | 1050 | 88 |
|  | 1100 | 100 |
| **PAAm-based microgels** | | |
| **nanogel type** | ***I* [mM]** | **Fraction of compartmentalized microgels [%]** |
| uncharged | 700 | 100 |
| negatively charged | 1000 | 37 |
| positively charged | 1750 | 100 |
| negatively + positively charged | 2000 | 100 |


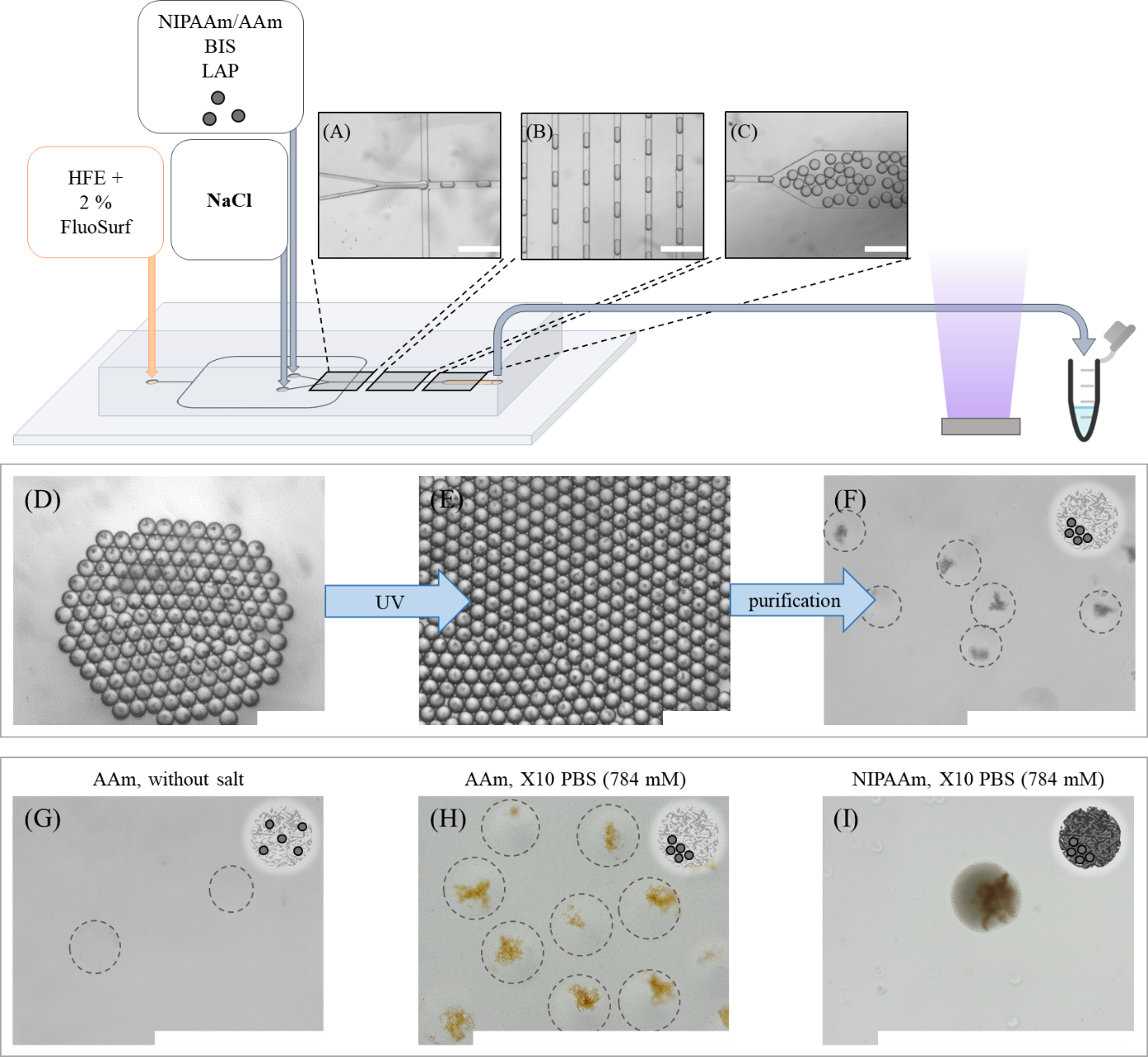


**Figure S6.** Droplet-based microfluidic set-up for the production of microgels with metal nanoparticle (schematically represented as grey spheres) compartments. Optical microscopy images show (D) aqueous droplets collected after the tubing, (E) emulsion after UV irradiation and (F) microgels containing metal nanoparticle compartments after purification and dispersed in water. Image (G) shows microgels with homogeneously distributed metal nanoparticles that were produced by using HPLC-grade water in the second aqueous phase compared to images (H) and (I) that show compartmentalized microgels in coloured brightfield microscopy based on AAm and NIPAAm, respectively. All scale bars represent 500 µm.

As further proof for the broad applicability of the introduced method, microgels with compartmentalized iron oxide nanoparticles (EMG 700) were fabricated. These nanoparticles are decorated with a negative surface charge for electrostatic stabilization. Thus, an increase in ionic strength is expected to screen the charges and lead to precipitation of the particles. For this, the same microfluidic set-up was used as presented before with few alterations. Firstly, instead of the nanogel dispersion, water was used to dissolve NIPAAm or AAm, BIS and LAP before adding only few microliters of the nanoparticle dispersion. Secondly, because of the dark colour and therefore high light absorbance of the nanoparticles, the emulsion was irradiated continuously in the tubing instead of in a vial to ensure complete UV irradiation and polymerization of every single droplet.

As second aqueous solution, either HPLC-grade water for homogeneous distribution of nanoparticles or X10 PBS buffer for compartmentalized nanoparticles were used. X10 PBS buffer has a salt concentration of 1567 mM resulting in an ionic strength of approximately 784 mM in the droplets. In contrast to the NiM-C where the nanogels precipitate rapidly on the microfluidic chip in the mixing section, here the nanoparticles take a longer time and only precipitate in the microfluidic tubing behind the outlet. After polymerization of the surrounding microgel network, the metal nanoparticle compartments are immobilized in the microgels, as seen by the darker areas in optical microscopy or brown areas in colored optical microscopy.


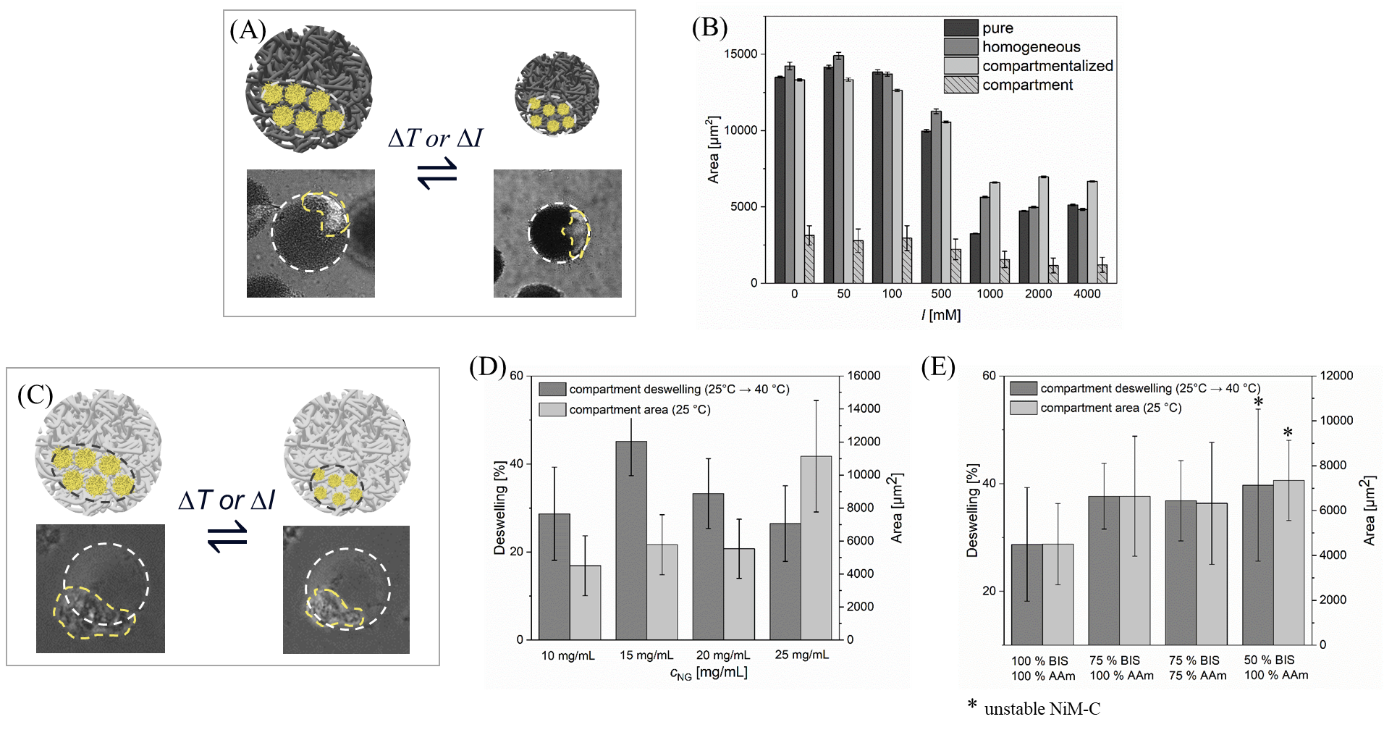


**Figure S7.** (A) Schematic and microscopy images of stimuli-responsive behavior of PNIPAAm-based NiM-C. (B) Ionic strength-responsive behavior of PNIPAAm-based microgels without nanogels, with homogeneous nanogel distribution and compartmentalized nanogels, as well as the compartment itself with *n* = 50 microgels measured for microgel area and *n* = 25 for compartment area. (C) Schematic and microscopy images of stimuli-responsive behavior of PAAm-based NiM-C. Measurements of the compartment size at 25 °C and deswelling when heating from 25 °C to 40 °C depending on nanogel concentration for *n* = 10 microgels (D), and BIS and AAm content for *n* = 10 microgels (E). All graphs show the average and respective standard deviation.

The microscopy images in **(A)** show PNIPAAm-based NiM-C with uncharged PNIPAAm-based nanogel compartments. In contrast to the PAAm-based NiM-C (**Figure 3**), these colloids do not exhibit anisotropic shape change as both compartments are similarly responsive to temperature and ionic strength. This is shown in **(B)**: The microgel itself decreases from around 13300 µm^2^ in area at low ionic strengths to approximately 6700 µm^2^ at high ionic strengths, while the compartment area decreases form 3100 µm^2^ to 1200 µm^2^. Therefore, the microgel decreases to around 50 % and the compartment to around 40 % of its original size. Both are very similar to each other, so the overall shape of the compartmentalized NiM-C stays the same.

For PAAm-based NiM-C with uncharged nanogel compartments, the anisotropic shape change is apparent in **Figure S7** **(C)** as the compartments deswell while the surrounding microgel stays at a constant size. This effect is investigated regarding variation of nanogel concentration **(D)**, and BIS and AAm content **(E)**. First, the nanogel concentration was varied between 10 mg  mL^‑1^ and 25 mg mL^-1^. Unexpectedly, the compartments do not increase linearly in size, which might indicate that they exhibit varying densities. However, while the overall trend indicates that an increase in concentration yields larger compartments, the large standard deviations must be considered.

Next, several samples with 10 mg mL^-1^ of the uncharged nanogels were synthesized with varying amounts of crosslinker and monomer in regards to the samples shown before. Here, a clear correlation between compartment size and deswelling is demonstrated. With decreasing crosslinker concentration, the microgel network becomes softer and the nanogel compartment increases in size while decreasing in density. Thus, the deswelling is more pronounced as well. When comparing the samples that contain 75 % BIS and either 100 % or 75 % AAm, nearly no differences can be recognized in either compartment size or deswelling. The monomer concentration does not seem to influence the microgel network density enough to have a noticeable influence on the compartment. For the last sample with only 50 % BIS it is important to note that the microgels were not stable anymore, but the compartments were intact after purification and could be measured nonetheless. Again, the compartment size and deswelling keep increasing. In addition to nanogel concentration, the crosslinker concentration can be tuned to not only adjust the compartment size but also its responsivity.


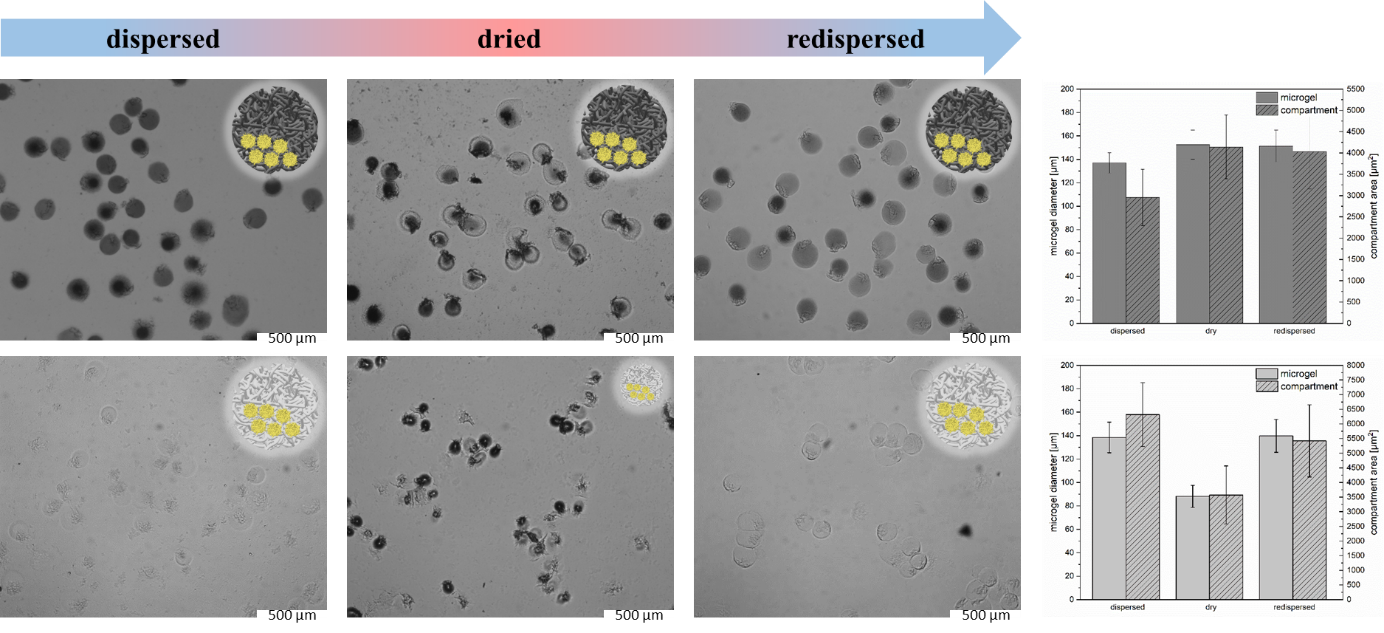


**Figure S8.** Study of drying and reswelling behavior of PNIPAAm-based (top) and PAAm-based (bottom) NiM-C using optical microscopy. Graphs on right-hand side show average diameters of microgels and average areas of compartments as well as their standard deviations for *n* = 50 microgels in the dispersed, dried and redispersed state.

PNIPAAm-based and PAAm-based NiM-C were fully dried over a period of several hours at room temperature. Subsequently, the gels were redispersed in HPLC-grade water and left to equilibrate for around 30 minutes. The NIPAAm-based NiM-C stay at approximately the same size even in the dried stage. However, the microscopy image shows that the microgels are less homogeneous due to the drying. Interactions between the microgels and glass slide may be a reason for the unaltered diameter of the microgels. After redispersing in water, the microgels return to their more homogeneous morphology as proven by the graph. The microgels can be fully redispersed after drying, but exhibit a slightly larger size when redispersed.

The PAAm-based NiM-C behave as expected: Both the PAAm network and the PNIPAAm-based nanogel compartments significantly decrease in size in the dried state and reswell to approximately their original size and spherical shape when redispersed in water.


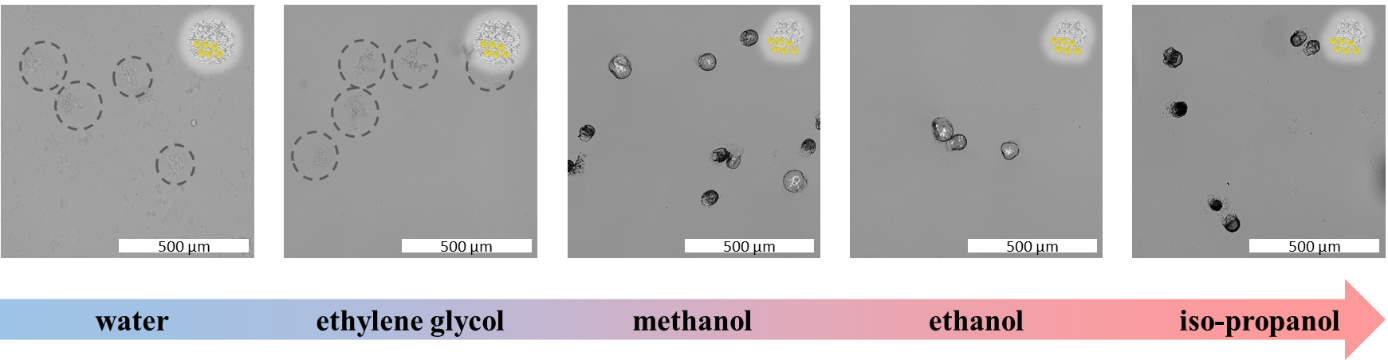


**Figure S9.** Optical microscopy images of PAAm-based NiM-C in a series of solvents with decreasing polarity.

The optical microscopy images reflect the findings from **Figure 3**. The PAAm NiM-C remain swollen in both water and ethylene glycol, the polar solvents. In methanol, the NiM-C significantly decrease in size and the exhibit a slight decrease in size towards less polar solvents, ethanol and isopropanol. The PNIPAAm nanogel compartments stay at an approximately constant size. The deformation of the compartments confirms that the slight decrease in area of the compartments results from compression that the surrounding network exerts on the internalized compartments.


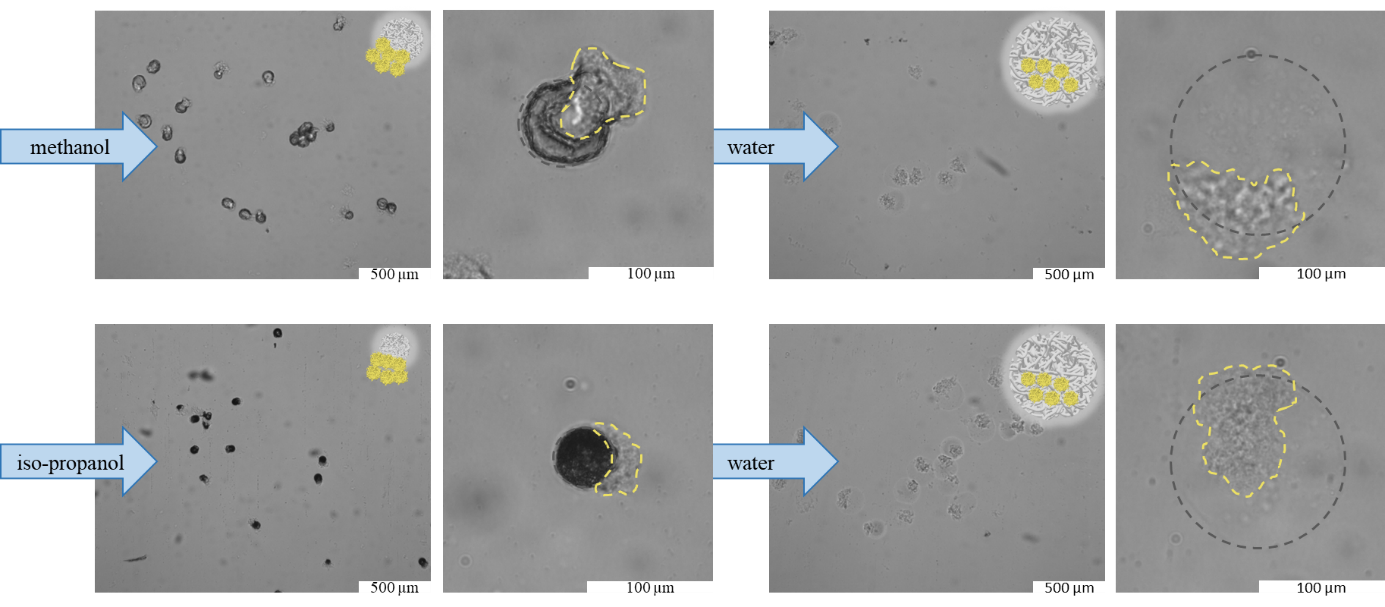


**Figure S10.** Optical microscopy images of PAAm-based NiM-C dispersed in organic solvents, methanol and iso-propanol, and redispersed in water.

The PAAm-based NiM-C were dispersed in methanol and iso-propanol, and redispersed in water afterwards. In methanol, the PAAm network significantly collapses due to its highly hydrophilic properties, while the PNIPAAm-based nanogel compartment stays swollen, resulting in an anisotropic particle. In iso-propanol, this effect is even more pronounced with even smaller microgel diameters and a darker color of the NiM-C due to the expulsion of water. In both cases, the NiM-C fully reswell when redispersed in water and regain their symmetric and spherical shape.


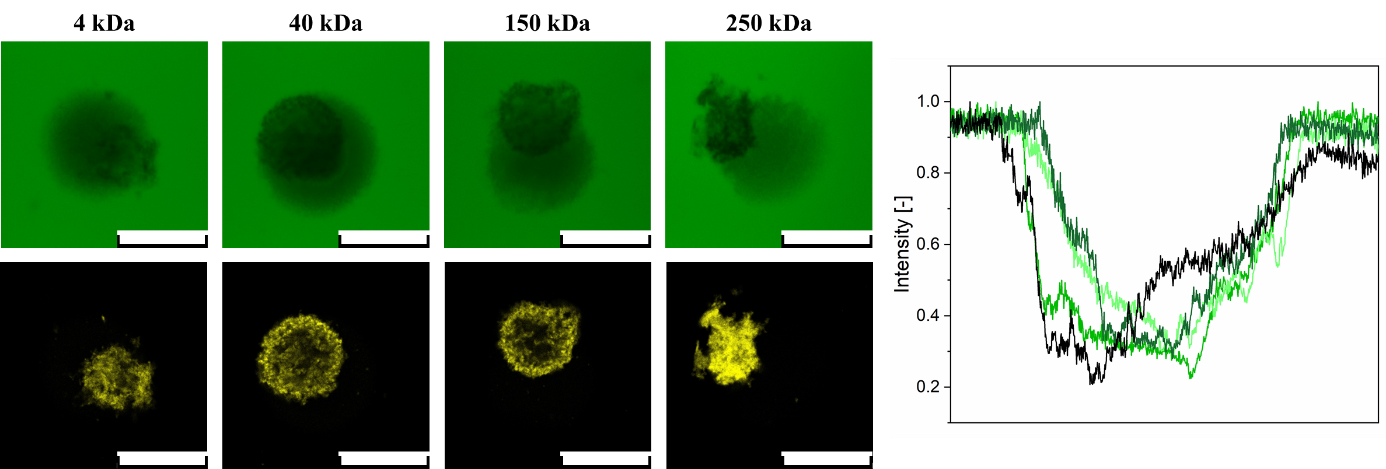
**Figure S11.** Permeability assay of compartmentalized PNIPAAm NiM-C produced at an ionic strength of 1000 mM to check reproducibility of permeability assay of compartmentalized PNIPAAm NiM-C produced at 500 mM. Green (top row) shows FITC-dextrans and yellow (bottom row) shows rhodamine B used for labelling of nanogels.

**Figure S11** shows the permeability assay for another compartmentalized PNIPAAm sample that contains double the salt concentration than the previous sample (**Figure 4**), 1000 mM compared to 500 mM. In addition, fluorescence of the rhodamine B labelled nanogels was detected to confirm that nanogel compartments are less permeable than the surrounding network. These measurements confirm reproducibility of the synthesis, measurement and results.


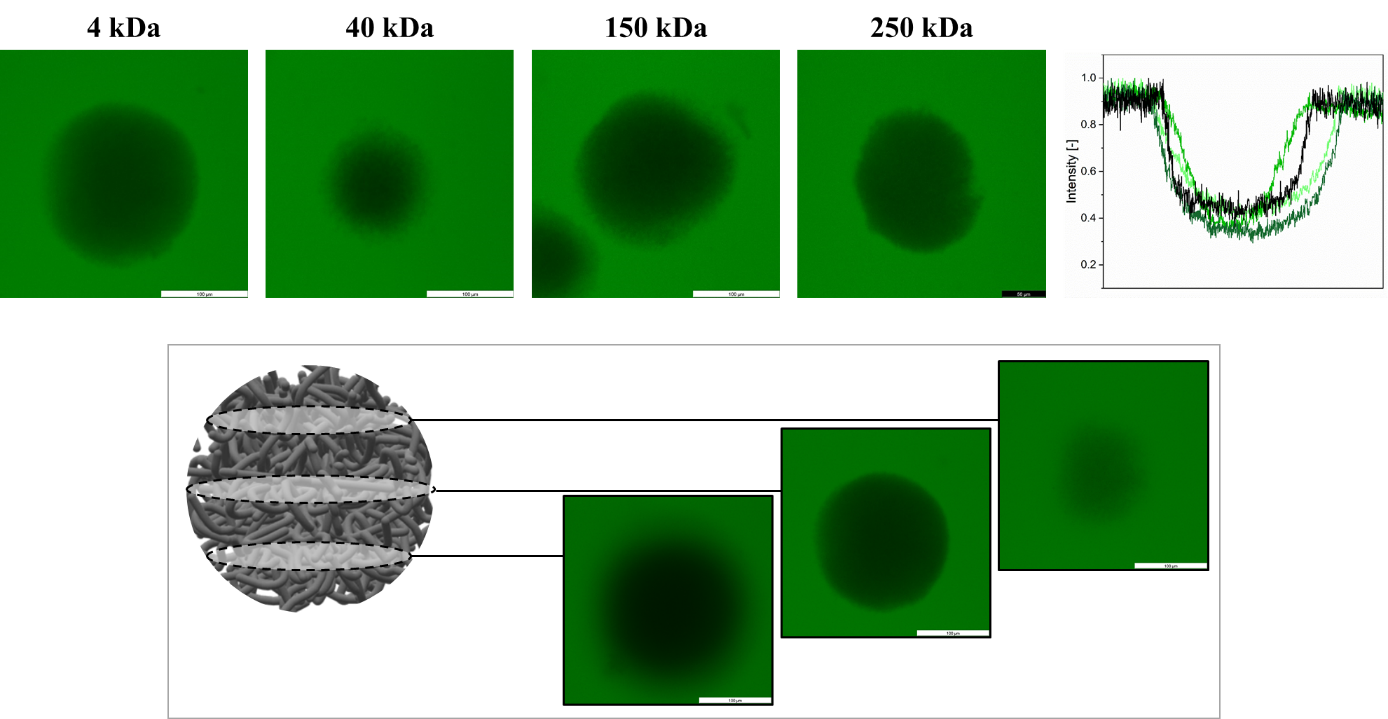
To investigate, whether the higher permeability of the compartmentalized NiM-C compared to the pure or homogeneous NiM-C results from nanogel incorporation or the ionic strength during synthesis, a reference sample of PNIPAAm microgels without nanogels but with an ionic strength of 1000 mM during the synthesis was produced.

**Figure S12.** Permeability assay of pure PNIPAAm microgels synthesized in the presence of 1000 mM NaCl as reference for previous experiments (top) and CLSM images of a z-stack (bottom). Scale bars represent 100 µm.

**Figure S12** depicts the reference sample of PNIPAAm microgels produced at high ionic strength without the incorporation of nanogels. Similar to what has been shown for the compartmentalized PNIPAAm-based samples (**Figure 4**), the microgels produced with an increased ionic strength present during the synthesis are more permeable than their counterparts produced in HPLC-grade water. This can be explained by the ionic strength-responsive behavior of linear and crosslinked PNIPAAm polymer chains: As the PNIPAAm chains for during UV irradiation and polymerization, they start to undergo phase transition and precipitate. This leads to higher porosity of the resulting particles.


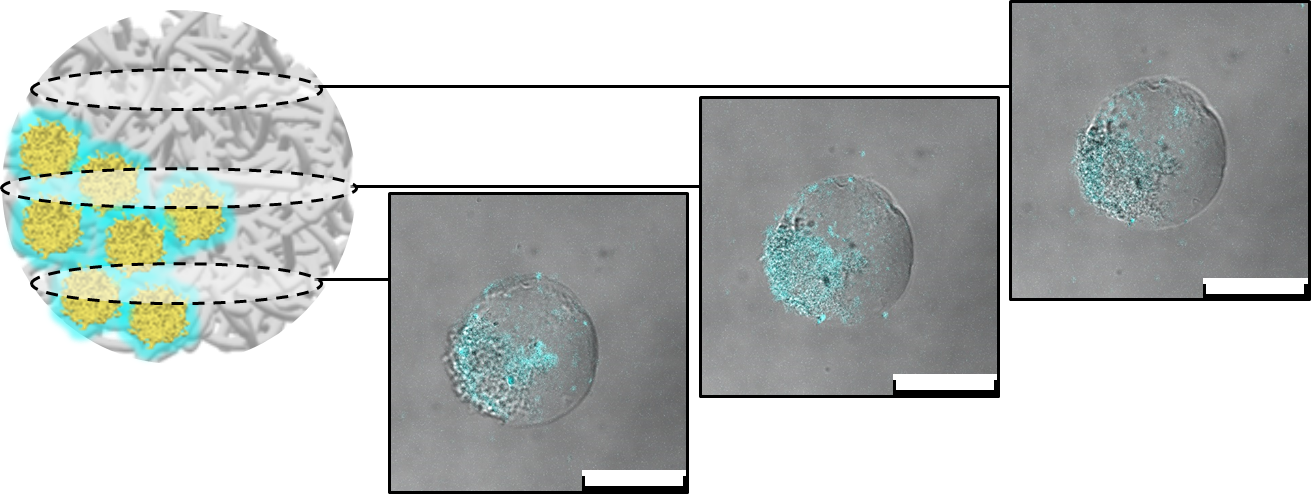


**Figure S13.** Overlay images of three different sections of the compartmentalized PAAm-based NiM-C that was used for uptake of the hydrophobic and fluorescent curcumin. Curcumin fluorescence is shown in cyan. Scale bars represent 100 µm.

The z-stack (**Figure S13**) confirms that the hydrophobic model drug curcumin is present throughout the complete hydrophobic PNIPAAm nanogel-containing compartment.


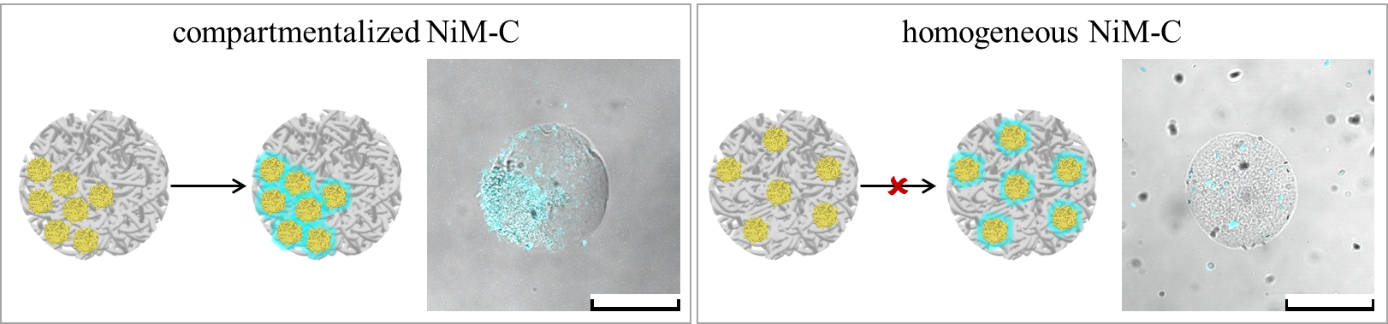


**Figure S14.** Schematic representation and CLSM overlay images for the uptake of curcumin into compartmentalized NiM-C (left) and homogeneous NiM-C (right).

**Figure S14** shows that uptake of curcumin is only successful for compartmentalized NiM-C. In case of homogeneous NiM-C only small agglomerates of curcumin are visible in the CLSM overlay image. The PNIPAAm-based nanogels must be compartmentalized in order to form a hydrophobic pocket in the microgel network without diminishing its properties.


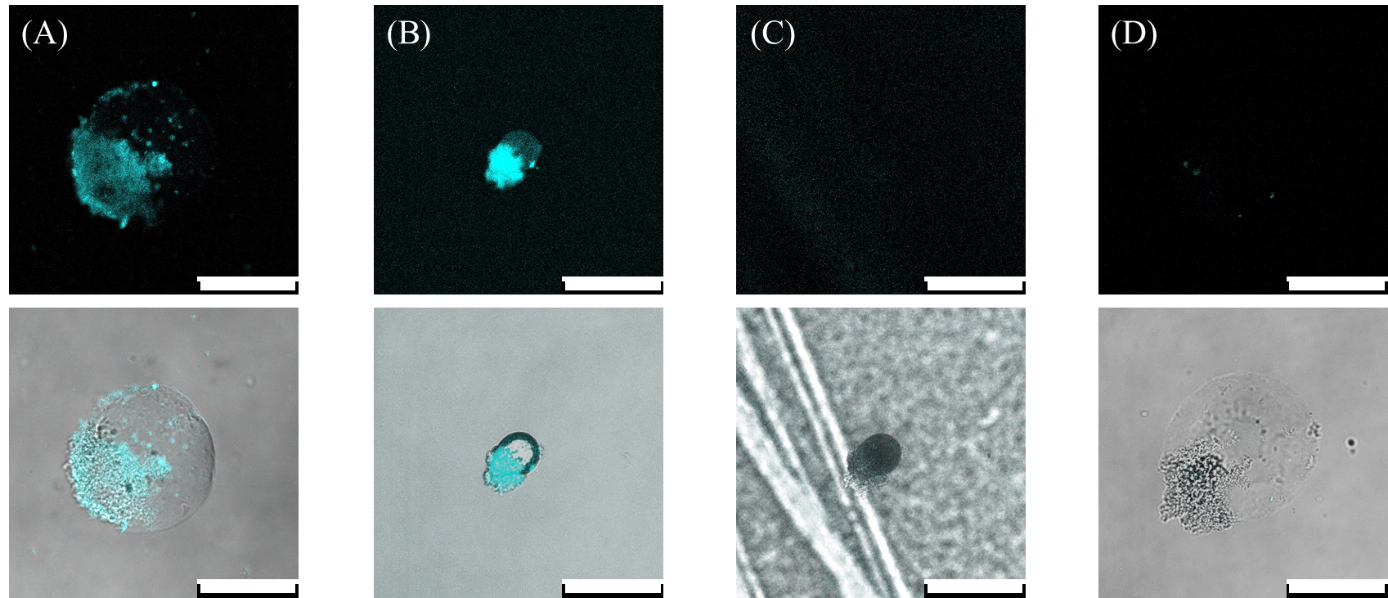


**Figure S15.** Fluorescence images (top row) and overlay images (bottom row) of compartmentalized PAAm-based NiM-C used for release experiments. (A) NiM-C after uptake of curcumin in water shows fluorescence only in the compartment. (B) After addition of some methanol, the NiM-C is in a water/methanol mixture leading to the collapse of the PAAm network. (C) After adding an excess of methanol, the NiM-C is fully collapsed and the curcumin is released. (D) NiM-C redispersed in water after release of curcumin.

After the uptake of curcumin into PAAm-based NiM-C, methanol was added to the aqueous dispersion to trigger the release of the model drug. When only little methanol is added (**Figure S15** **(B)**), the solution can be assumed to be composed of both water and methanol. As seen in **Figure 3**, the PAAm network collapses in the less polar environment. By adding an excess of methanol (**Figure S15** **(C)**), the network fully collapses and the curcumin is released into the surrounding solvent. Lastly, the NiM-C was redispersed in water (**Figure S15** **(D)**) and the absence of fluorescence in the compartment confirms the successful release.

**References**

[1] H. F. Mathews, M. I. Pieper, S.-H. Jung, A. Pich, *Angew. Chem., Int. Ed.* **2023**, 62, e202304908.

**Author Contributions**

M. I. Pieper and H. F. Mathews developed the concept and planned the experiments with the support of Prof. Dr. A. Pich. M. I. Pieper performed the synthetic experiments and carried out the CLSM experiments. M. I. Pieper recorded and analyzed microscopy images. M. I. Pieper designed the graphics. M. I. Pieper wrote the manuscript with the help of H. F. Mathews and Prof. Dr. A. Pich. All authors reviewed this manuscript and approved of the final version.
